# Supplementary material for: Systematic evaluation of fMRI data-processing pipelines for consistent functional connectomics
Source: Nat Commun. 2024 Jun 4;15:4745. doi: 10.1038/s41467-024-48781-5 (PMC11150439; doi:10.1038/s41467-024-48781-5)
Supplement: Supplementary file 1 — Supplementary Information [file 41467_2024_48781_MOESM1_ESM.pdf]

Supplementary Materials for

Systematic evaluation of fMRI data-processing pipelines for consistent functional connectomics

Luppi *et al.*

Guide to pipeline selection in the *Pipeline Selection Tool (Supplementary Data 2)*

This document provides a guide for the use of the interactive pipeline selection tool (Supplementary Data 2). The tool is in the form of an Excel file which allows the user to filter pipelines based on specific user-defined criteria. Pipelines can be filtered based on multiple criteria combined to allow the user to specify preferred preconditions for a pipeline choice. The criteria for pipeline selection:

- **Criterion (I):** Avoiding spurious differences ("PDiv ranking"). Since the two networks that we consider are derived from different scans of the same healthy individuals under conditions in which no experimentally meaningful changes in functional network topology are expected, we aim to identify pipelines that minimise test-retest PDiv. We consider pipelines as candidates for optimal if they are in the top 20% in terms of the global PDiv rank calculated across all four test-retest intervals.
- **Criterion (II):** Detecting true experimental differences ("propofol"). Suitable pipelines should detect a significant effect for propofol, in the right direction, in both propofol datasets, i.e., a pipeline is excluded if it fails to detect the expected effect in either of the two propofol datasets.
- **Criterion (III):** Detecting inter-individual differences ("within-between"). A pipeline fails this criterion if the resulting networks are more similar between than within subjects more than 50% of the times, for any of the three test-retest datasets.
- **Criterion (IV):** Avoiding motion-induced differences ("motion"). A pipeline fails this criterion if its PDiv has a significant correlation with differences in head motion in any of the three test-retest datasets.
- **Criterion (V):** Non-empty networks. As a final sanity check, we also exclude any pipelines that remove all connections from a network, in any of the three test-retest datasets.

Column B identifies pipelines that pass all selection criteria (II-V above) and are within the top 20% of average PDiv ranks. The same can be found in Column AX when relaxing the PDiv criterion to 50%. Pipelines that fulfil all of these criteria can be selected by clicking the option "Selected" in the filter.

Combinations of multiple user-defined criteria can be obtained by selecting options in multiple filters at once. For instance, if the user wanted to identify all pipelines which fulfil the above five criteria, used a single scale parcellation type and no global signal regression, this is what the result would look like (showing one pipeline which fulfils these criteria):

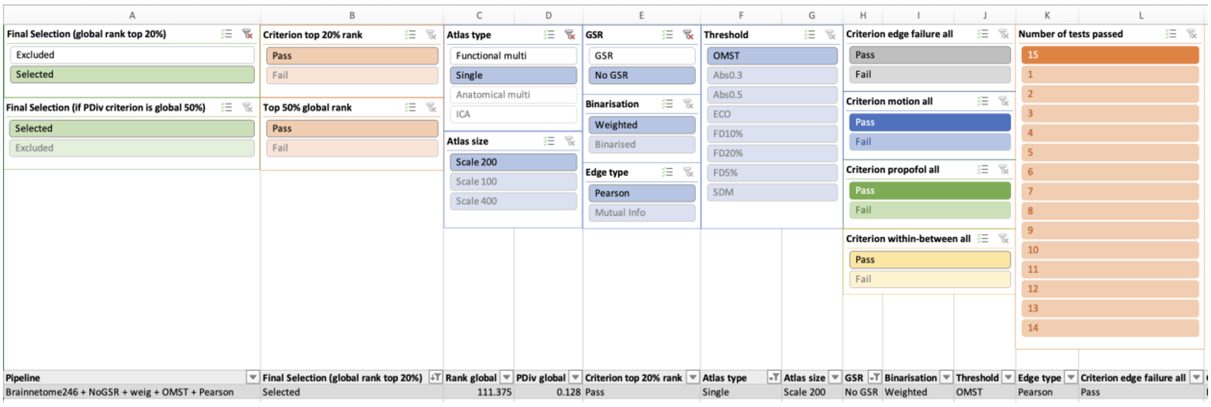

Pipeline	Final Selection (global rank top 20%)	Rank global	PDiv global	Criterion top 20% rank	Atlas type	Atlas size	GSR	Binarisation	Threshold	Edge type	Criterion edge failure all
Brainnetome246 + NoGSR + weig + OMST + Pearson	Selected	111.375	0.128	Pass	Single	Scale 200	No GSR	Weighted	OMST	Pearson	Pass

In contrast, if the user only cared about a pipeline passing Criteria II and V above, regardless of portrait divergence or pre-processing choices, the result may look as follows:

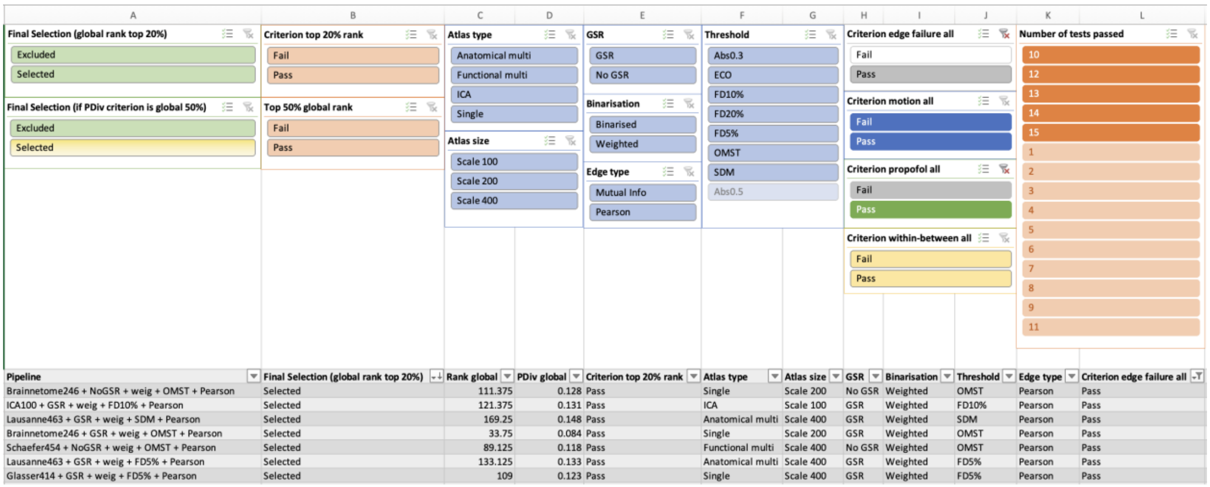

Pipeline	Final Selection (global rank top 20%)	Rank global	PDiv global	Criterion top 20% rank	Atlas type	Atlas size	GSR	Binarisation	Threshold	Edge type	Criterion edge failure all
Brainnetome246 + NoGSR + weig + OMST + Pearson	Selected	111.375	0.128	Pass	Single	Scale 200	No GSR	Weighted	OMST	Pearson	Pass
ICA100 + GSR + weig + FD10% + Pearson	Selected	121.375	0.131	Pass	ICA	Scale 100	GSR	Weighted	FD10%	Pearson	Pass
Lausanne463 + GSR + weig + SDM + Pearson	Selected	169.25	0.148	Pass	Anatomical multi	Scale 400	GSR	Weighted	SDM	Pearson	Pass
Brainnetome246 + GSR + weig + OMST + Pearson	Selected	33.75	0.084	Pass	Single	Scale 200	GSR	Weighted	OMST	Pearson	Pass
Schaefer454 + NoGSR + weig + OMST + Pearson	Selected	89.125	0.118	Pass	Functional multi	Scale 400	No GSR	Weighted	OMST	Pearson	Pass
Lausanne463 + GSR + weig + FD5% + Pearson	Selected	133.125	0.133	Pass	Anatomical multi	Scale 400	GSR	Weighted	FD5%	Pearson	Pass
Glasser414 + GSR + weig + FD5% + Pearson	Selected	109	0.123	Pass	Single	Scale 400	GSR	Weighted	FD5%	Pearson	Pass

In this example, for the threshold slicer, option Abs0.5 can now no longer be selected because no pipelines with this pre-processing choice fulfil the propofol and non-empty network criteria.

A reset can be achieved by clicking on the filter icon with the red cross in the upper right corner of a given filter panel.

If the user wanted to include multiple options in a given filter panel (for instance if all pipelines with parcellation scale 200 and 400 were to be selected), the first option should be selected, followed by a click + command (or right click) on the second option. This would yield the following:

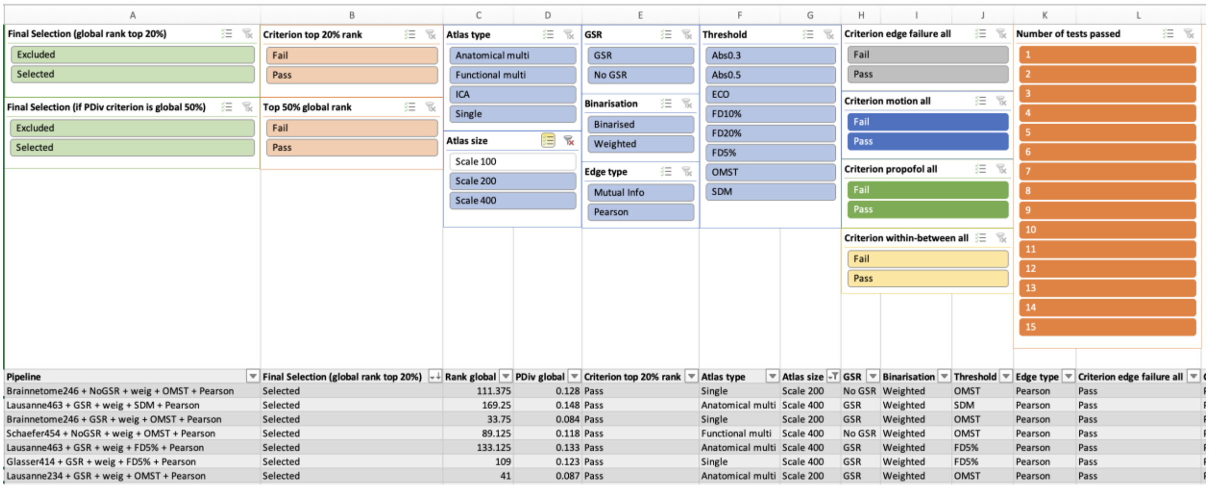

Pipeline	Final Selection (global rank top 20%)	Rank global	PDiv global	Criterion top 20% rank	Atlas type	Atlas size	GSR	Binarisation	Threshold	Edge type	Criterion edge failure all
Brainnetome246 + NoGSR + weig + OMST + Pearson	Selected	111.375	0.128	Pass	Single	Scale 200	No GSR	Weighted	OMST	Pearson	Pass
Lausanne463 + GSR + weig + SDM + Pearson	Selected	169.25	0.148	Pass	Anatomical multi	Scale 400	GSR	Weighted	SDM	Pearson	Pass
Brainnetome246 + GSR + weig + OMST + Pearson	Selected	33.75	0.084	Pass	Single	Scale 200	GSR	Weighted	OMST	Pearson	Pass
Schaefer454 + NoGSR + weig + OMST + Pearson	Selected	89.125	0.118	Pass	Functional multi	Scale 400	No GSR	Weighted	OMST	Pearson	Pass
Lausanne463 + GSR + weig + FD5% + Pearson	Selected	133.125	0.133	Pass	Anatomical multi	Scale 400	GSR	Weighted	FD5%	Pearson	Pass
Glasser414 + GSR + weig + FD5% + Pearson	Selected	109	0.123	Pass	Single	Scale 400	GSR	Weighted	FD5%	Pearson	Pass
Lausanne234 + GSR + weig + OMST + Pearson	Selected	41	0.087	Pass	Anatomical multi	Scale 200	GSR	Weighted	OMST	Pearson	Pass

Alternatively, filtering and sorting of the data based on any column available in the excel sheet can be done by clicking the downward facing arrow next to a column name in row 2.

Supplementary Tables

Table S1. Parcellations adopted in the present study, by scale (rows) and method (columns).

	Anatomical multi-scale	Functional multi-scale	Single-scale	ICA
**Scale-100**	Lausanne 129	Schaefer 100 + Melbourne 16	AAL 90	100 components
**Scale-200**	Lausanne 234	Schaefer 200 + Melbourne 32	Brainnetome 246	200 components
**Scale-400**	Lausanne 463	Schaefer 400 + Melbourne 54	Glasser 360 + Melbourne 54	300 components

Table S2. Edge filtering schemes adopted in the present study.

Filtering Scheme	Description
Fixed Density 5% (FD5%)	Top 5% of strongest edges
Fixed Density 10% (FD10%)	Top 10% of strongest edges
Fixed Density 20% (FD20%)	Top 20% of strongest edges
Absolute Threshold 0.3 (Abs0.3)	Edges with value > 0.3
Absolute Threshold 0.5 (Abs0.5)	Edges with value > 0.5
Efficiency Cost Optimisation (ECO)	Average node degree = 3, to maximise trade-off between overall efficiency and wiring cost
Structural Density Matching (SDM)	Proportional thresholding, with same density as the HCP group-average DTI data parcellated using the same parcellation
Orthogonal Minimum Spanning Trees (OMST)	Optimisation of global efficiency minus wiring cost, by combining independent minimum spanning trees of the network.

Supplementary Figures

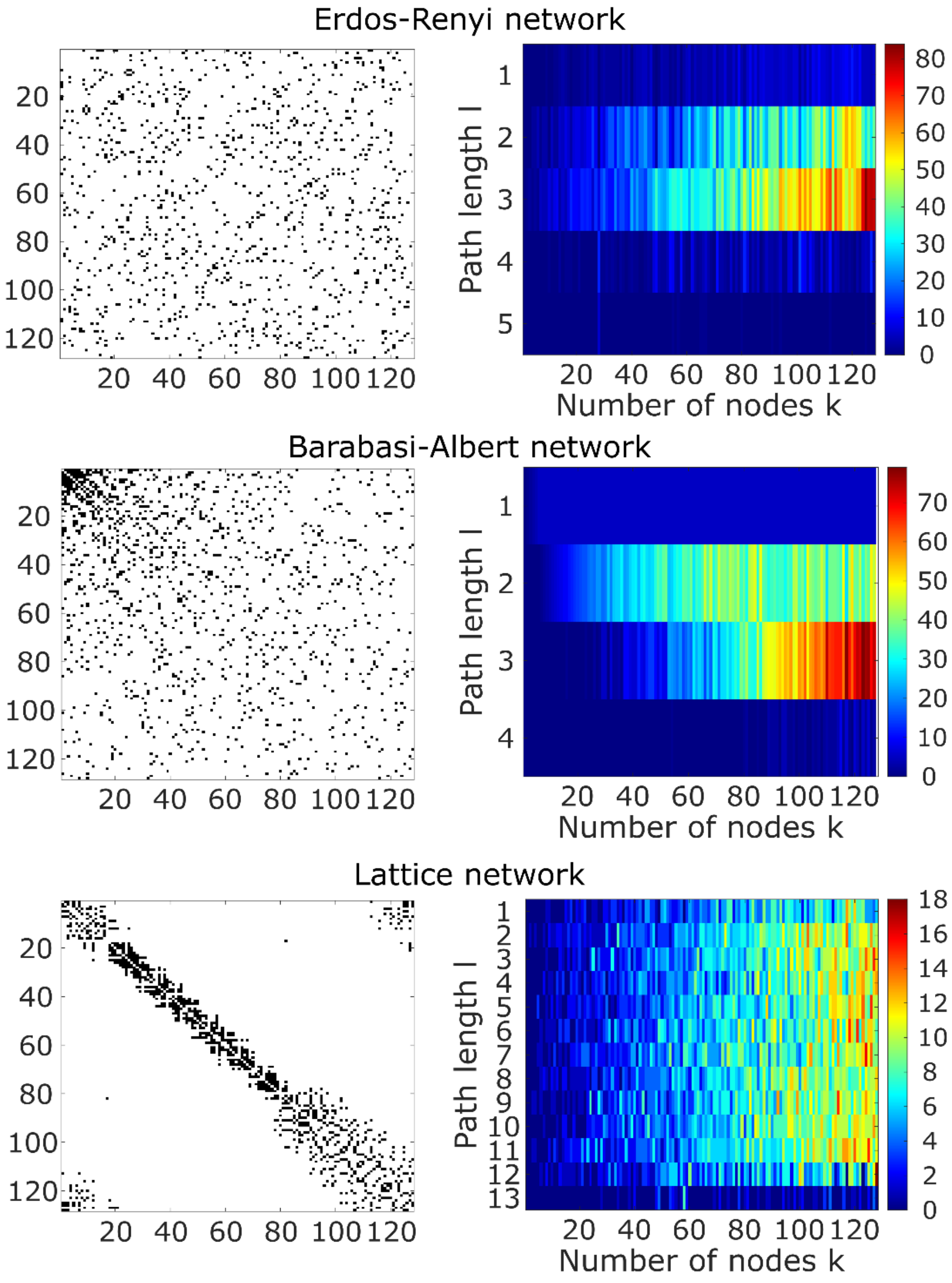

Figure S1. Example networks (left) and their portraits (right). From the top: Erdos-Renyi random network, Barabasi-Albert preferential attachment network, and lattice network. All networks are binary with an approximate density of 6%. A network portrait for a binary network is a matrix B whose rows each correspond to a histogram obtained by thresholding the matrix of shortest paths between the networks's constituent nodes, at each path length *l* between 0 and the network's diameter *L,* such that entry $B_{l,k}$ encodes the number of nodes that have *k* nodes at distance *l*. PDiv between ER and BA networks is 0.26; PDiv between ER and Lattice networks is 0.90; PDiv between BA and Lattice networks is 0.93.

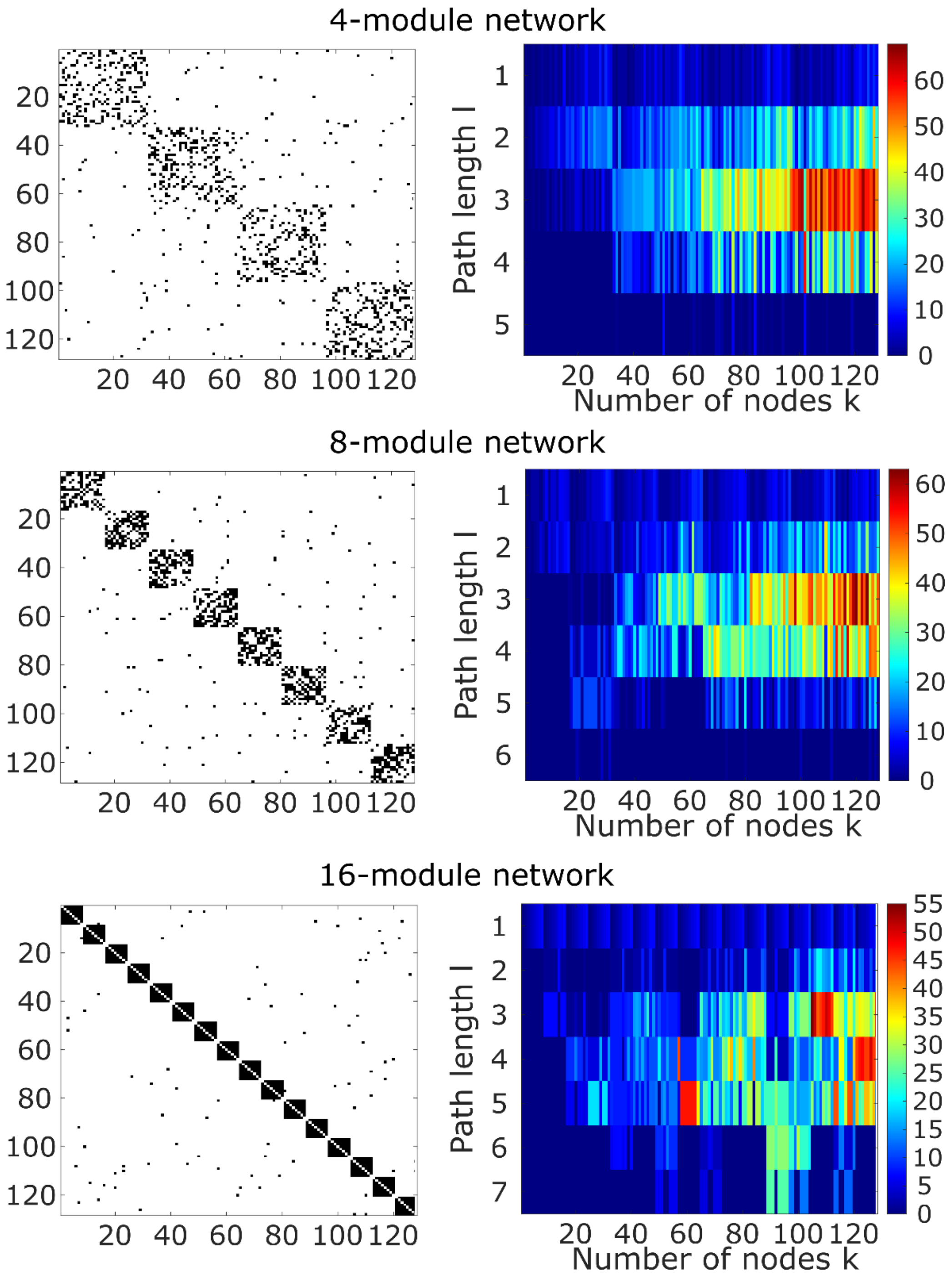

Figure S2. Additional examples of networks (left) and their portraits (right). From the top: modular networks with 4, 8, and 16 equal-sized modules, respectively. All networks are binary with an approximate density of 6%. A network portrait for a binary network is a matrix B whose rows each correspond to a histogram obtained by thresholding the matrix of shortest paths between the networks's constituent nodes, at each path length l between 0 and the network's diameter L, such that entry $B_{l,k}$ encodes the number of nodes that have k nodes at distance l. PDiv between the 4-module and 8-module networks is 0.36; PDiv between 8-module and 16-module networks is 0.52; PDiv between the 4-module and 16-module networks is 0.68. Note how the two most extreme cases (4 and 16 modules) have the largest PDiv, and how the modular organisation of each network is reflected in the first row of its network portrait.

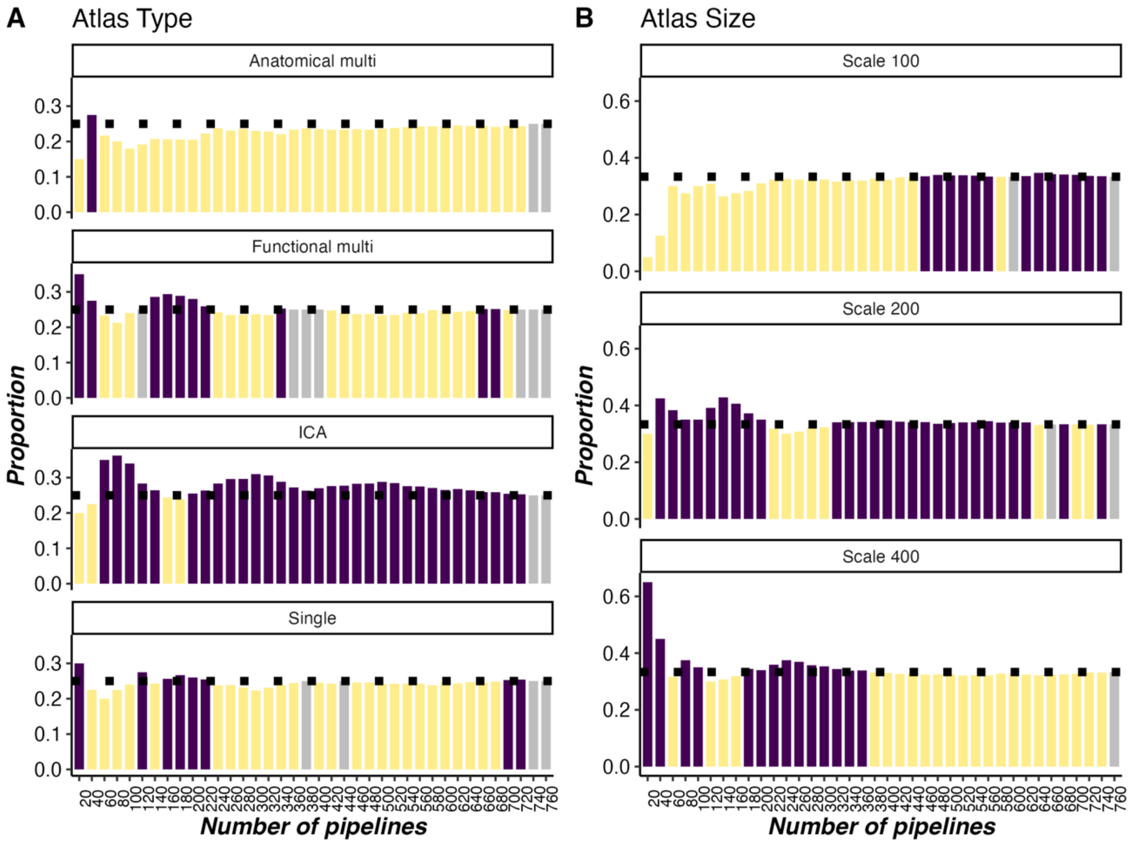

Figure S3. Progression of pipeline choices as a function of node definition and average PDiv across all datasets. (A) Divided by parcellation type (anatomical multi-scale, functional multi-scale, or single-scale). (b) By parcellation scale. With each subsequent bin, the next best 20 pipelines are added to calculate how many among this set of pipelines were constructed using each of the available options.

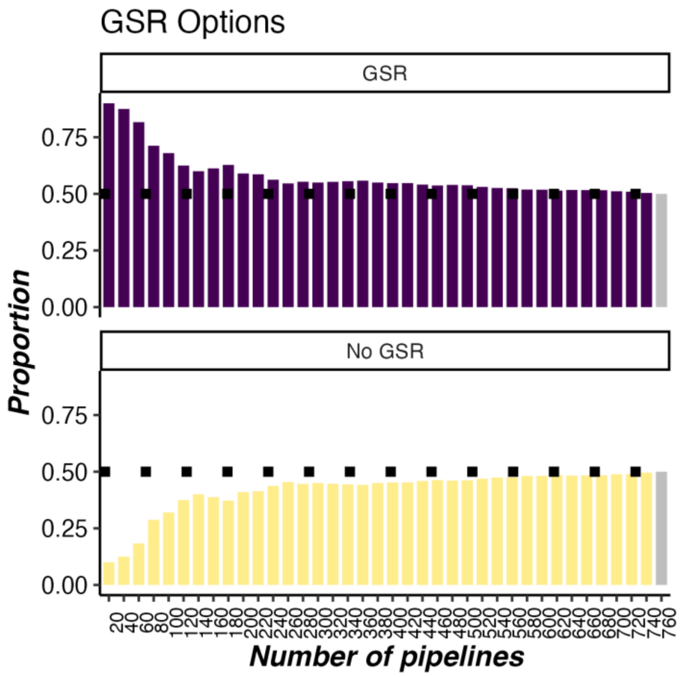

Figure S4. Progression of pipeline choices as a function of GSR use and average PDiv across all datasets. With each subsequent bin, the next best 20 pipelines are added to calculate how many among this set of pipelines were constructed using each of the available options.

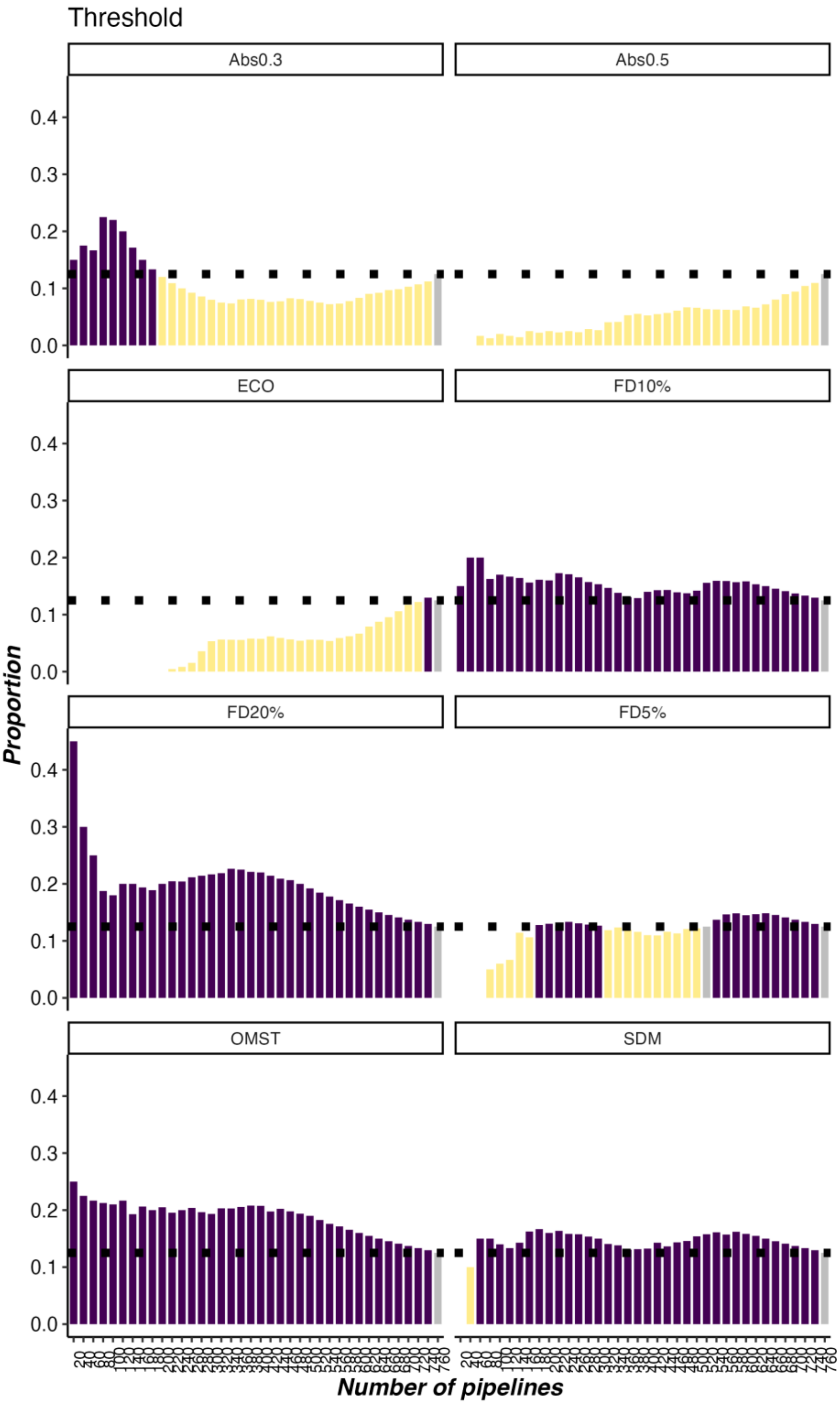

Figure S5. Progression of pipeline choices as a function of filtering scheme and average PDiv across all datasets. With each subsequent bin, the next best 20 pipelines are added to calculate how many among this set of pipelines were constructed using each of the available options.

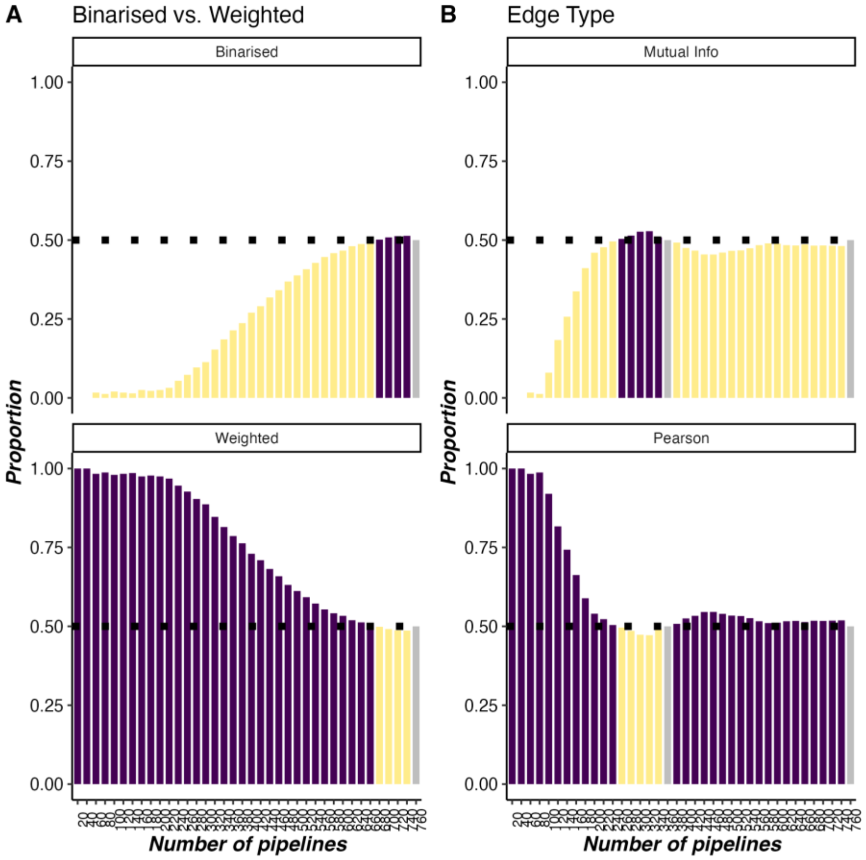

Figure S6. Progression of pipeline choices as a function of edge construction and average PDiv across all datasets. (A) Binary vs weighted edges. (B) Edges quantified in terms of mutual information or Pearson correlation. With each subsequent bin, the next best 20 pipelines are added to calculate how many among this set of pipelines were constructed using each of the available options.

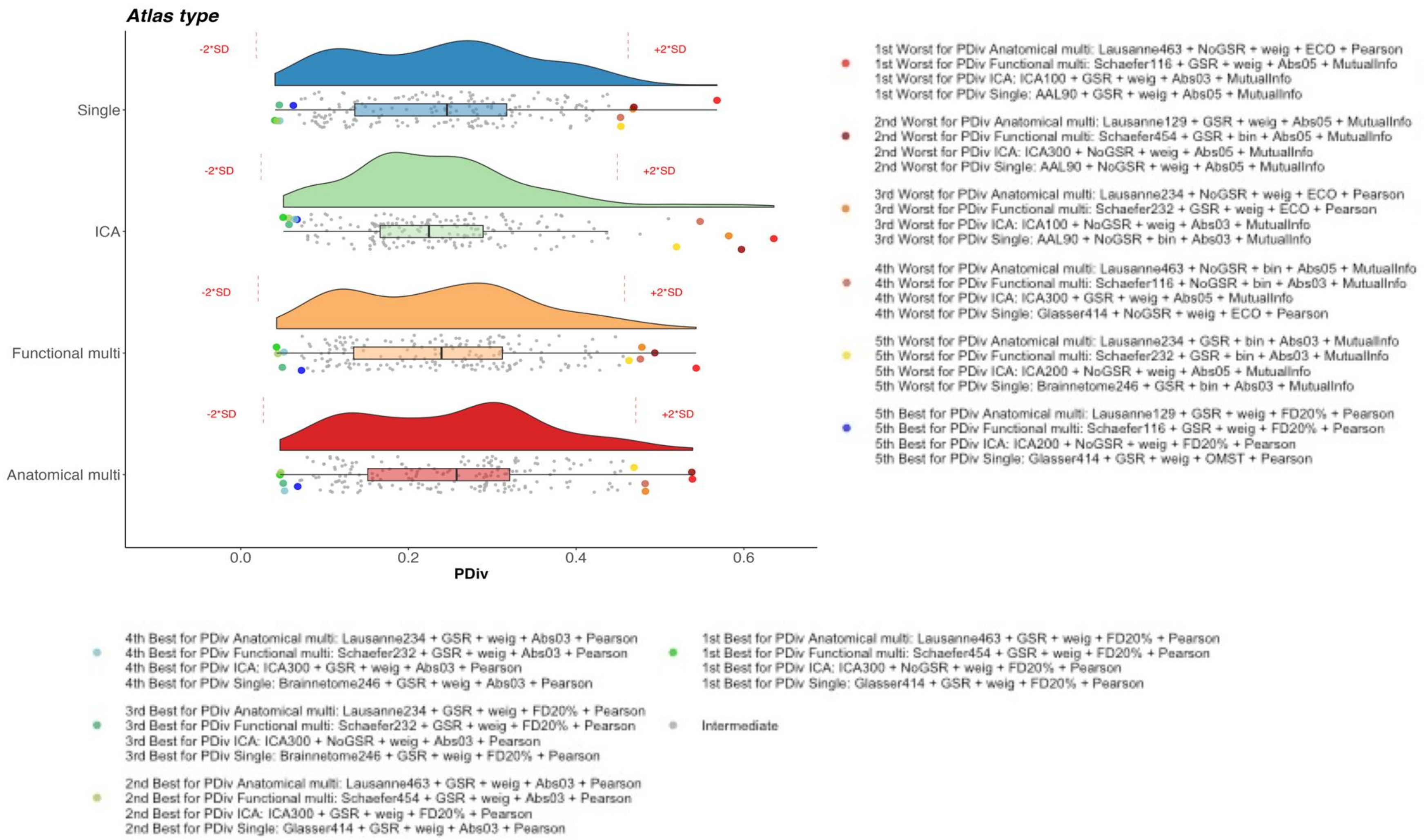

Figure S7. Portrait divergence (PDiv) by parcellation type – Cambridge test-retest dataset. Box-plot center line, median; box limits, upper and lower quartiles; whiskers, 1.5x interquartile range. Each data-point represents one pipeline (n=768).

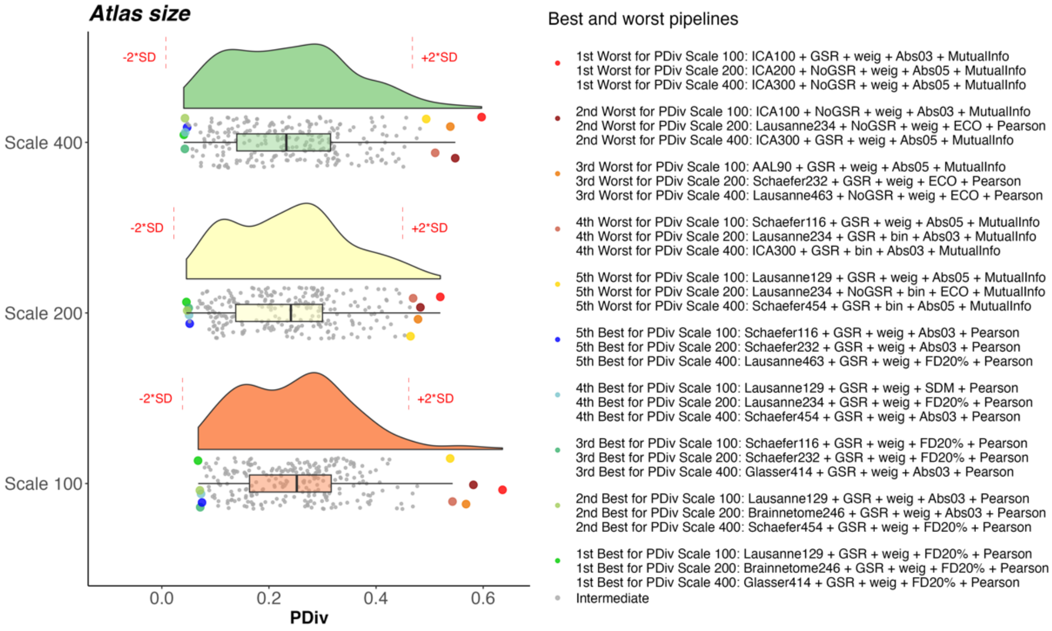

Figure S8. Portrait divergence (PDiv) by parcellation scale – Cambridge test-retest dataset. Box-plot center line, median; box limits, upper and lower quartiles; whiskers, 1.5x interquartile range. Each data-point represents one pipeline (n=768).

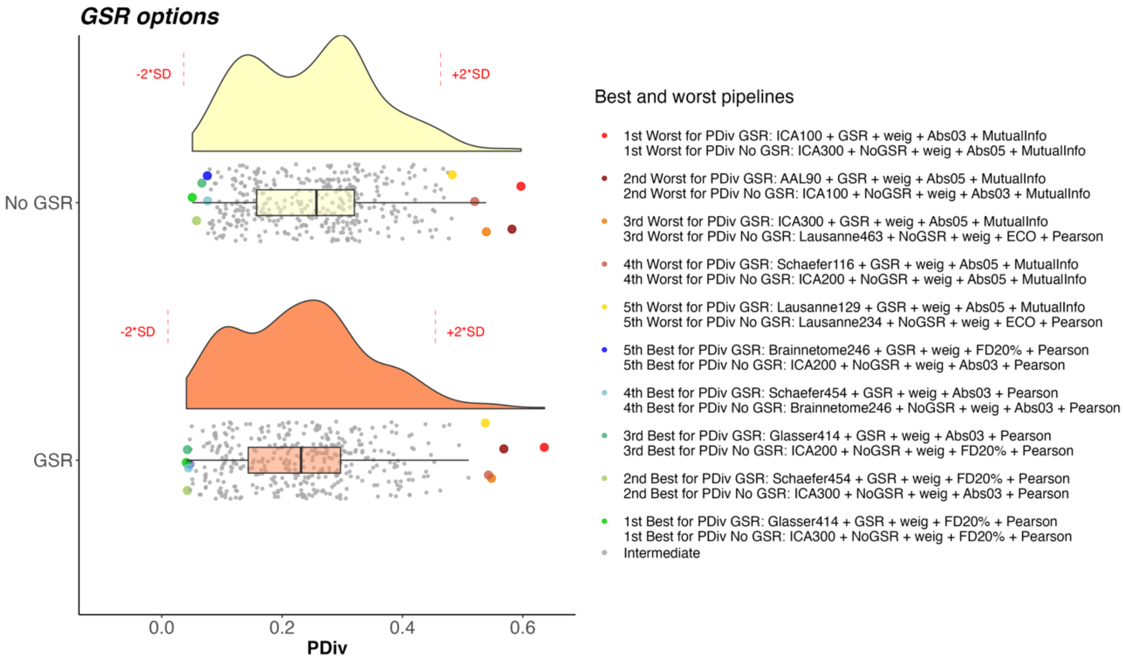

Figure S9. Portrait divergence (PDiv) by GSR use – Cambridge test-retest dataset. Box-plot center line, median; box limits, upper and lower quartiles; whiskers, 1.5x interquartile range. Each data-point represents one pipeline (n=768).

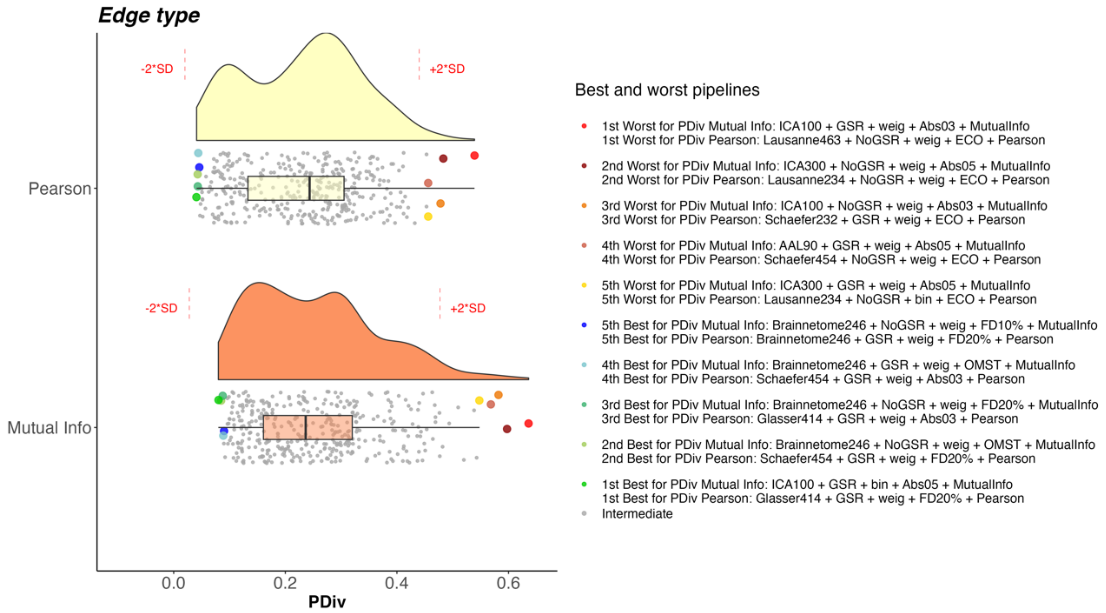

Figure S10. Portrait divergence (PDiv) by edge quantification method type – Cambridge test-retest dataset. Box-plot center line, median; box limits, upper and lower quartiles; whiskers, 1.5x interquartile range. Each data-point represents one pipeline (n=768).

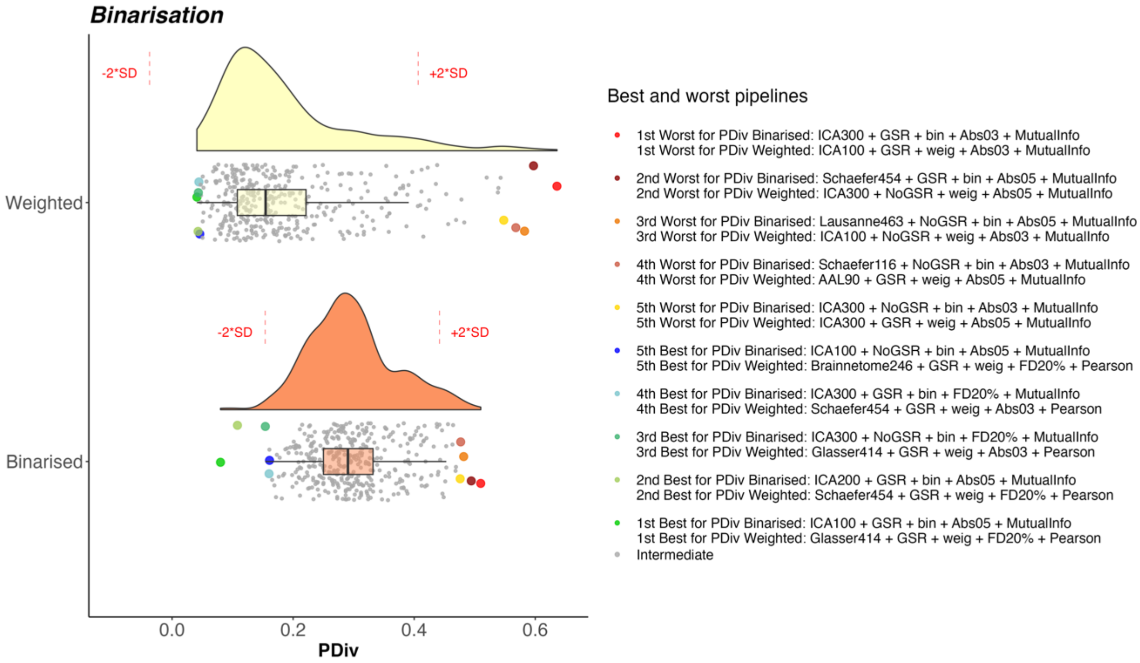

Figure S11. Portrait divergence (PDiv) by binarisation choice – Cambridge test-retest dataset. Box-plot center line, median; box limits, upper and lower quartiles; whiskers, 1.5x interquartile range. Each data-point represents one pipeline (n=768).

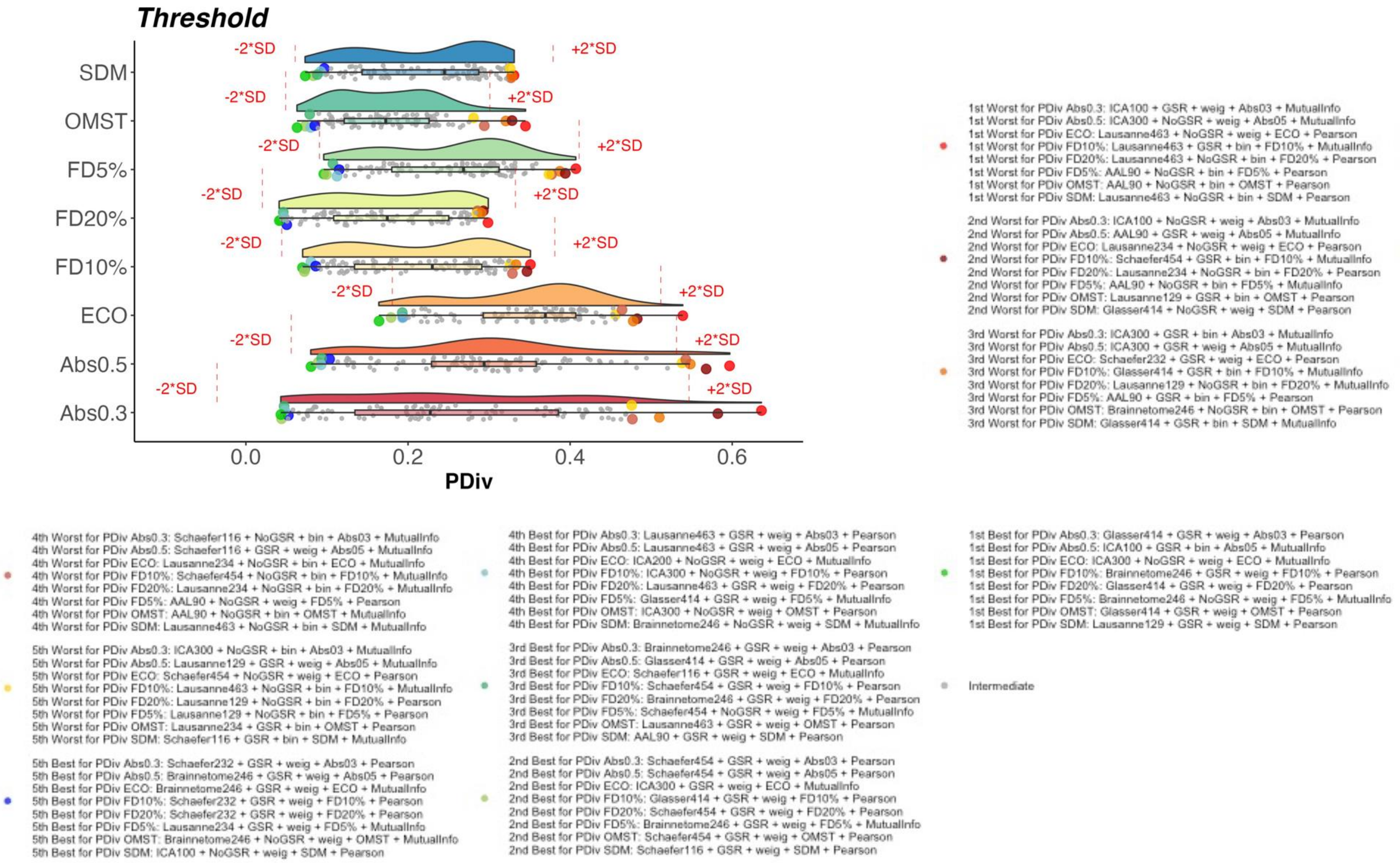

Figure S12. Portrait divergence (PDiv) by edge filtering method – Cambridge test-retest dataset. Box-plot center line, median; box limits, upper and lower quartiles; whiskers, 1.5x interquartile range. Each data-point represents one pipeline (n=768).

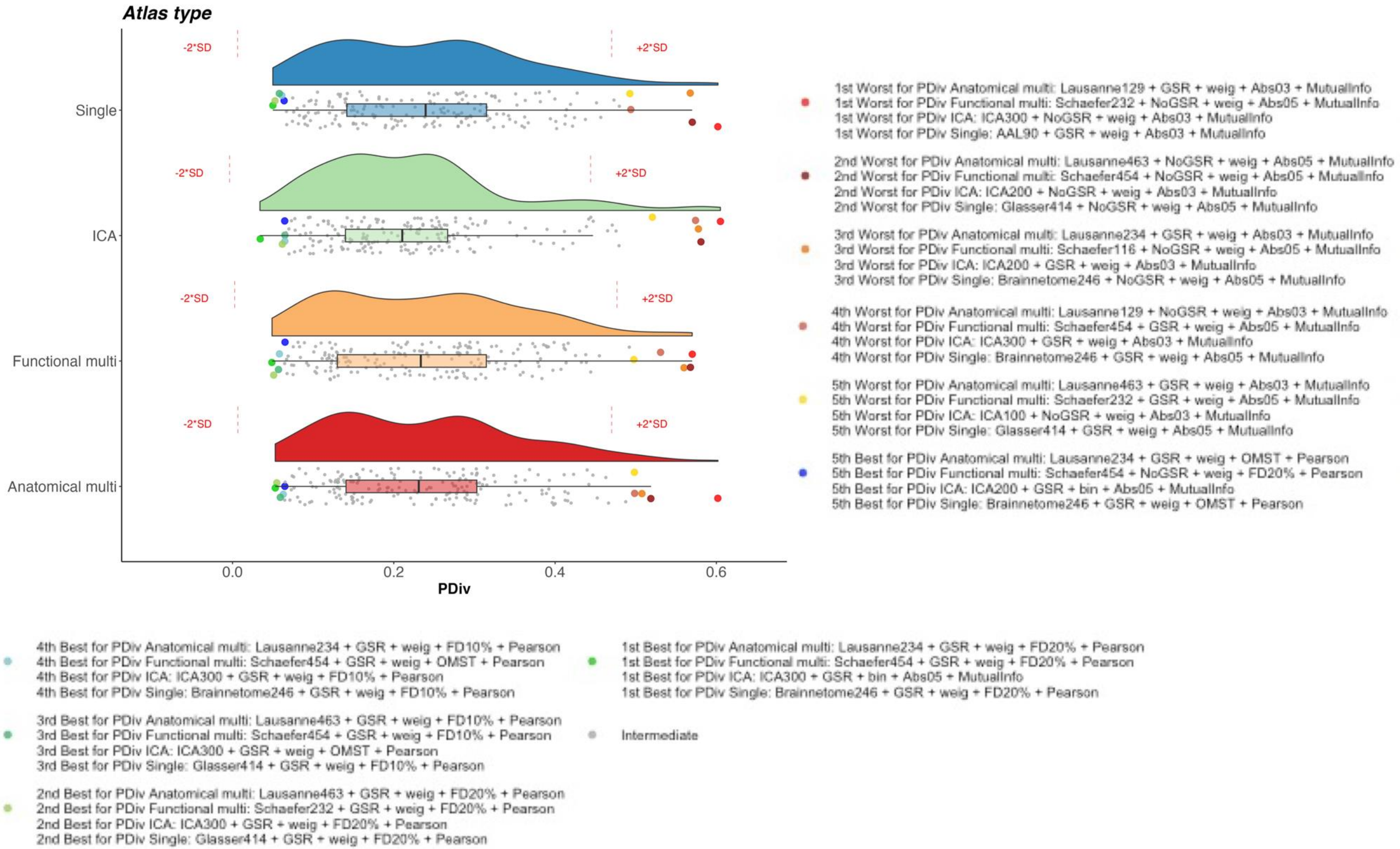

Figure S13. Portrait divergence (PDiv) by parcellation type – NYU short-term dataset. Box-plot center line, median; box limits, upper and lower quartiles; whiskers, 1.5x interquartile range. Each data-point represents one pipeline (n=768).

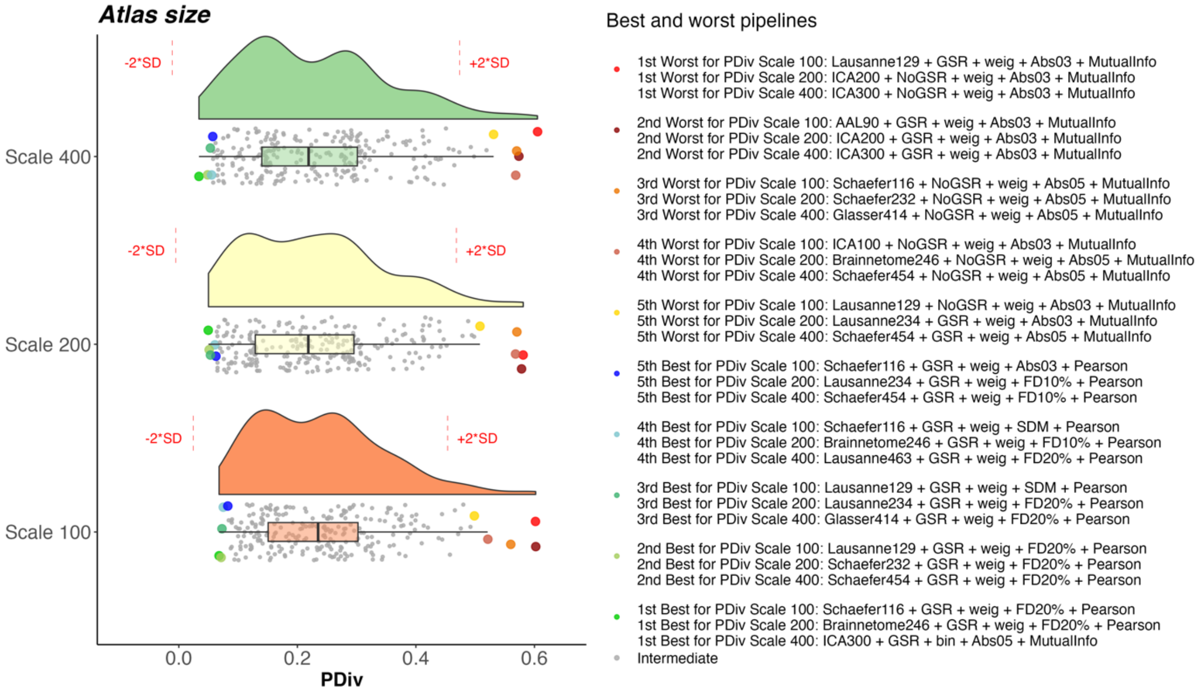

Figure S14. Portrait divergence (PDiv) by parcellation scale – NYU short-term dataset. Box-plot center line, median; box limits, upper and lower quartiles; whiskers, 1.5x interquartile range. Each data-point represents one pipeline (n=768).

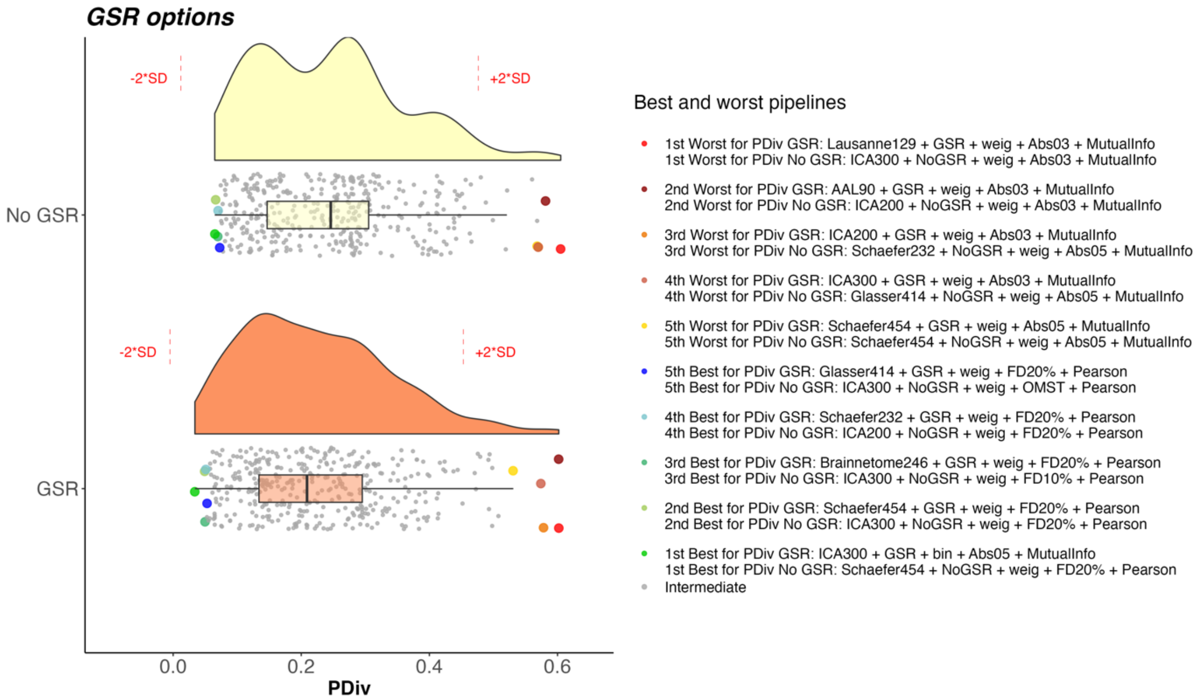

Figure S15. Portrait divergence (PDiv) by GSR use – NYU short-term dataset. Box-plot center line, median; box limits, upper and lower quartiles; whiskers, 1.5x interquartile range. Each data-point represents one pipeline (n=768).

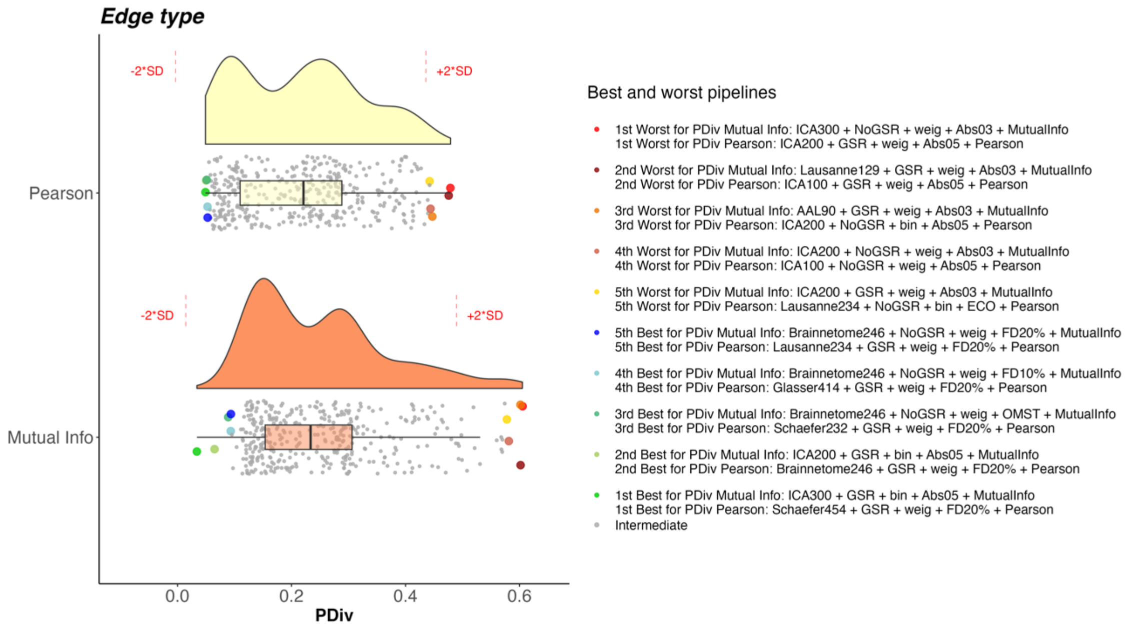

Figure S16. Portrait divergence (PDiv) by edge quantification method type – NYU short-term dataset. Box-plot center line, median; box limits, upper and lower quartiles; whiskers, 1.5x interquartile range. Each data-point represents one pipeline (n=768).

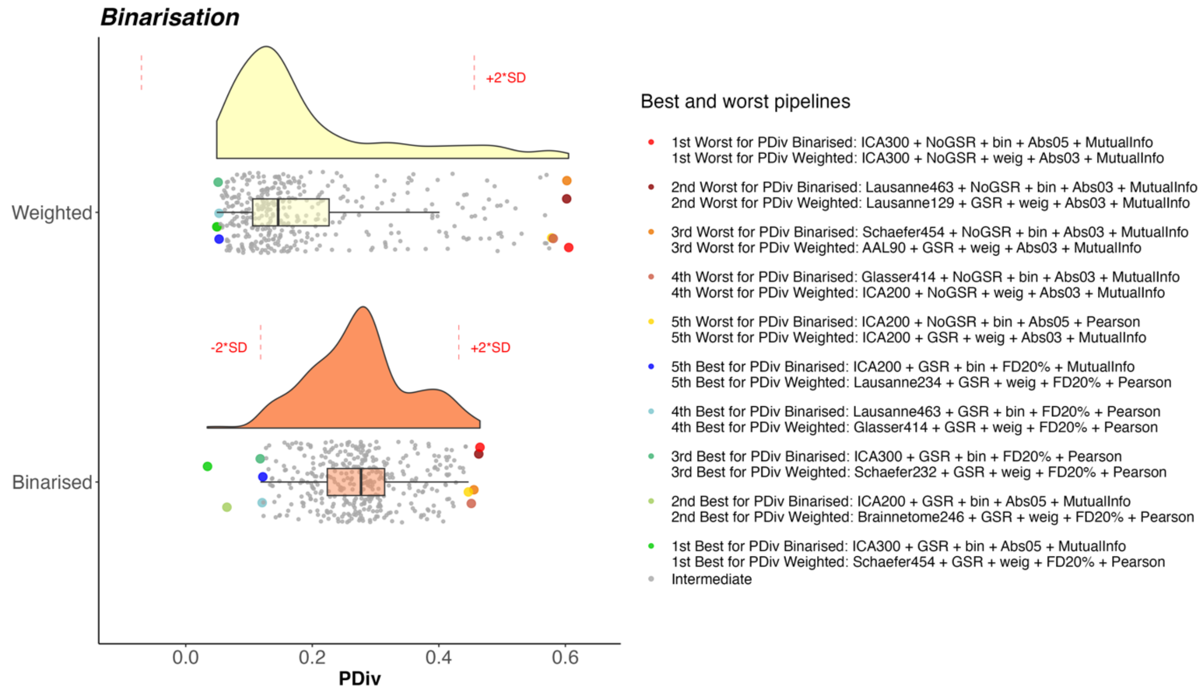

Figure S17. Portrait divergence (PDiv) by binarisation choice – NYU short-term dataset. Box-plot center line, median; box limits, upper and lower quartiles; whiskers, 1.5x interquartile range. Each data-point represents one pipeline (n=768).

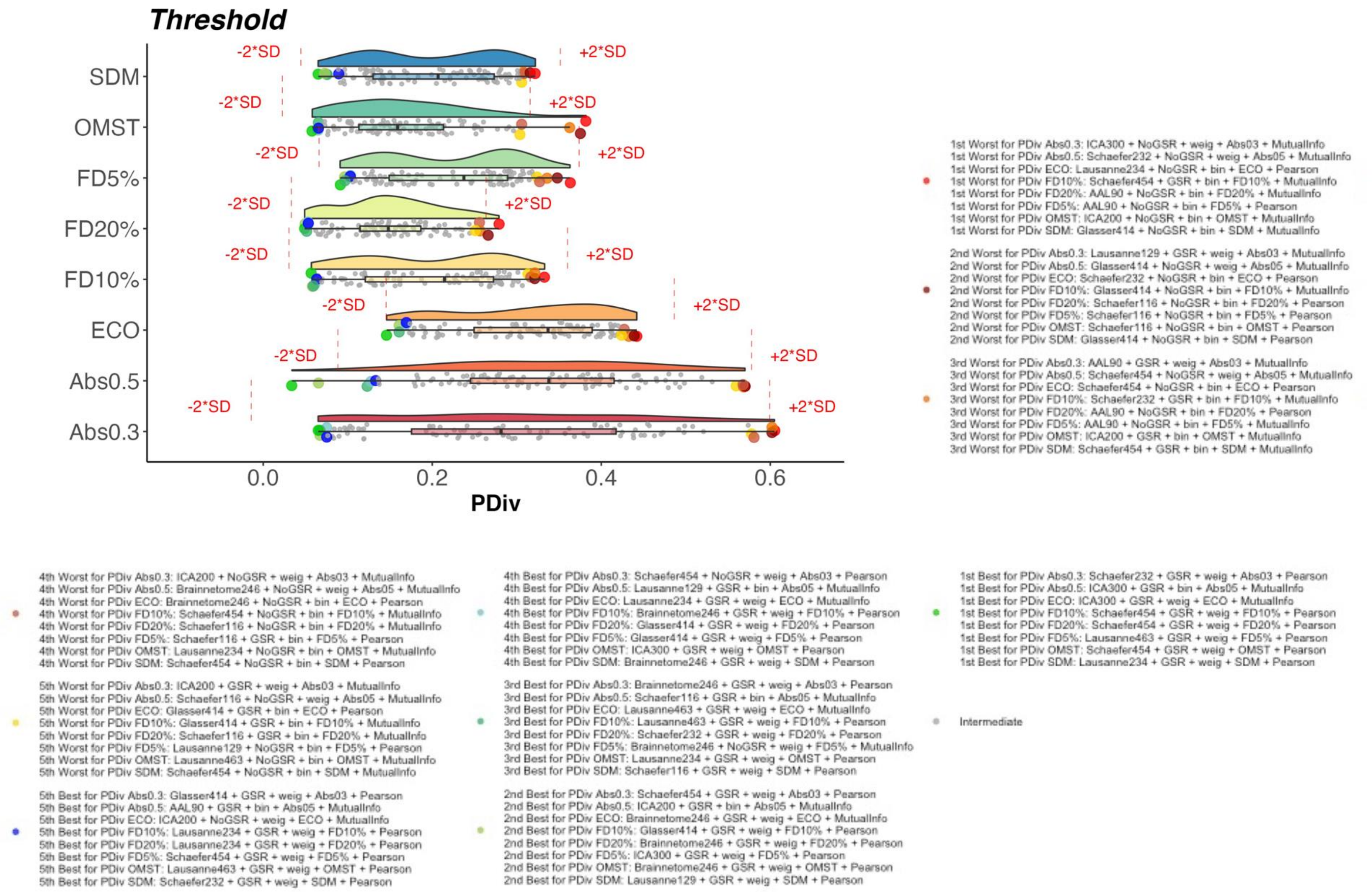

Figure S18. Portrait divergence (PDiv) by edge filtering method – NYU short-term dataset. Box-plot center line, median; box limits, upper and lower quartiles; whiskers, 1.5x interquartile range. Each data-point represents one pipeline (n=768).

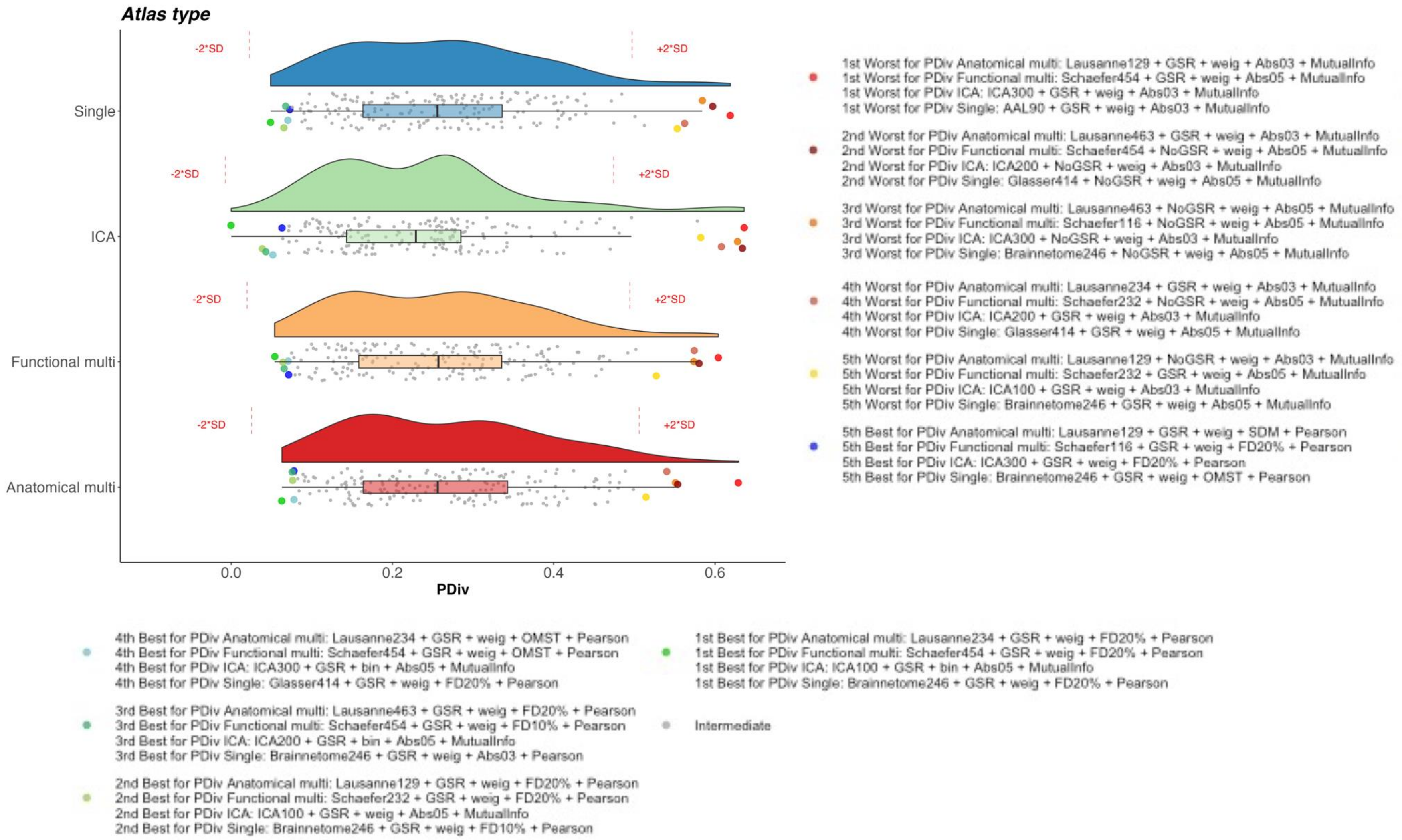

Figure S19. Portrait divergence (PDiv) by parcellation type – NYU long-term dataset. Box-plot center line, median; box limits, upper and lower quartiles; whiskers, 1.5x interquartile range. Each data-point represents one pipeline (n=768).

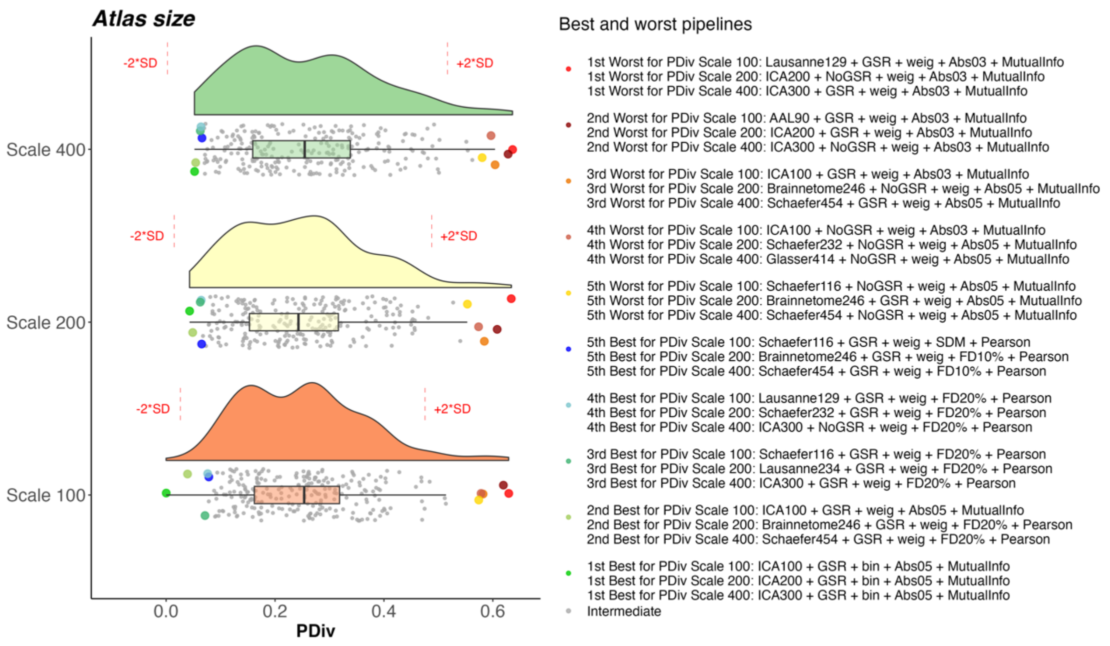

Figure S20. Portrait divergence (PDiv) by parcellation scale – NYU long-term dataset. Box-plot center line, median; box limits, upper and lower quartiles; whiskers, 1.5x interquartile range. Each data-point represents one pipeline (n=768).

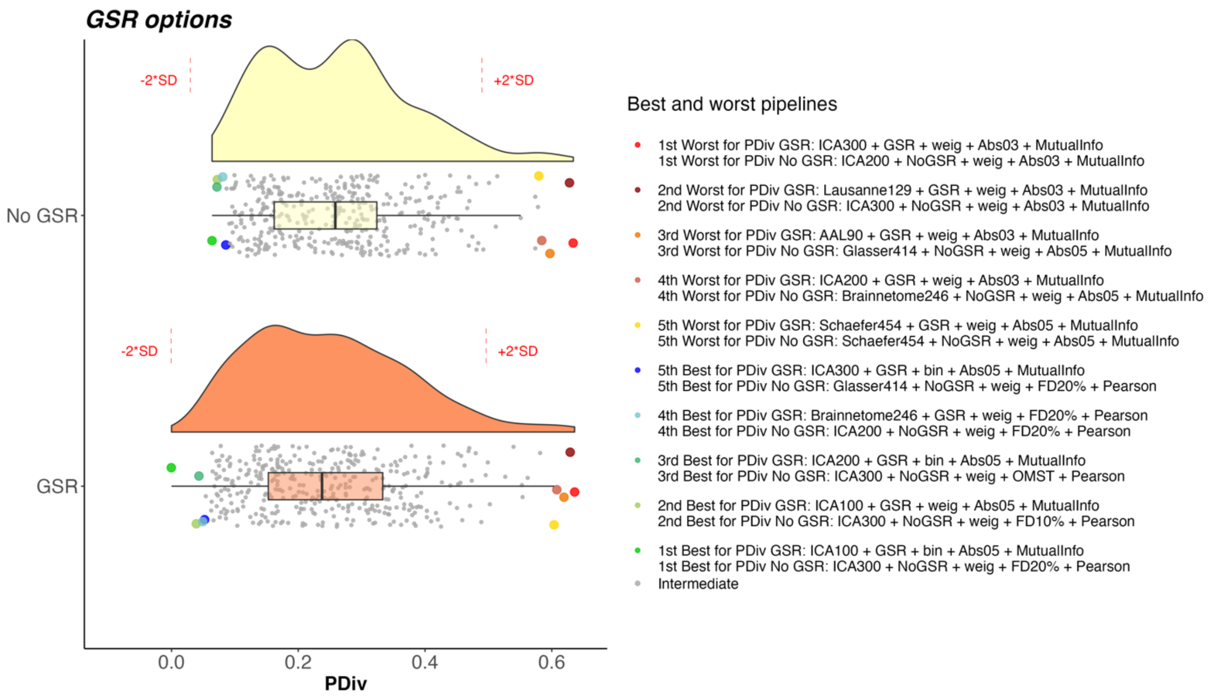

Figure S21. Portrait divergence (PDiv) by GSR use – NYU long-term dataset. Box-plot center line, median; box limits, upper and lower quartiles; whiskers, 1.5x interquartile range. Each data-point represents one pipeline (n=768).

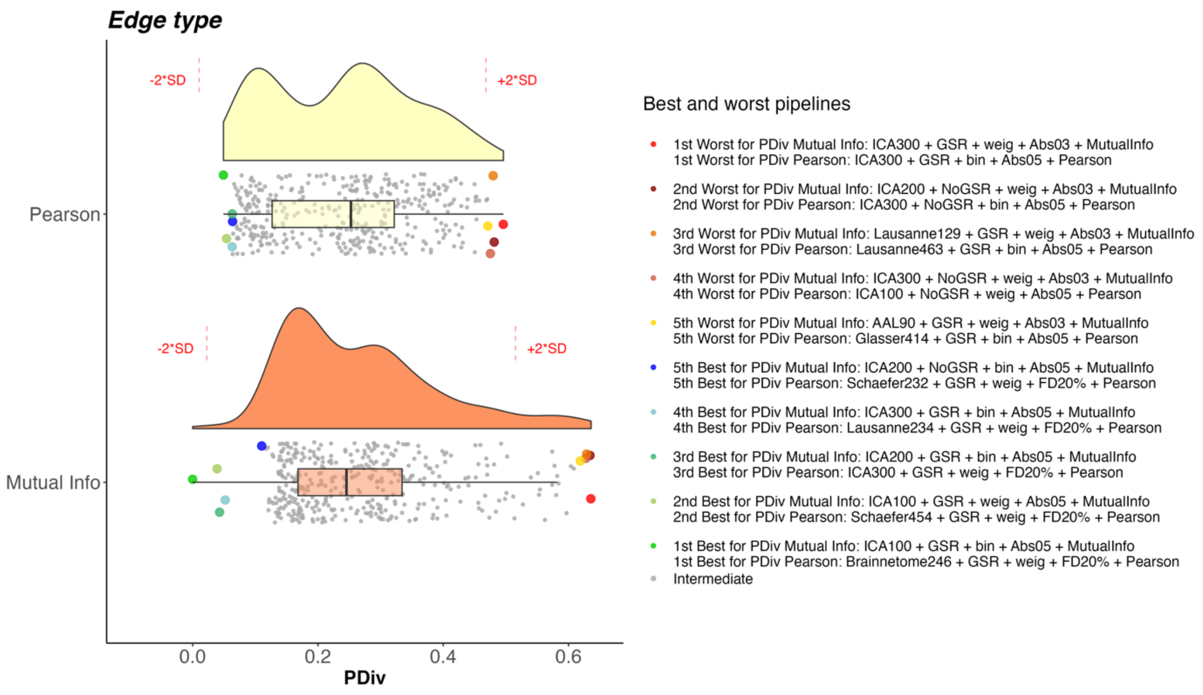

Figure S22. Portrait divergence (PDiv) by edge quantification method type – NYU long-term dataset. Box-plot center line, median; box limits, upper and lower quartiles; whiskers, 1.5x interquartile range. Each data-point represents one pipeline (n=768).

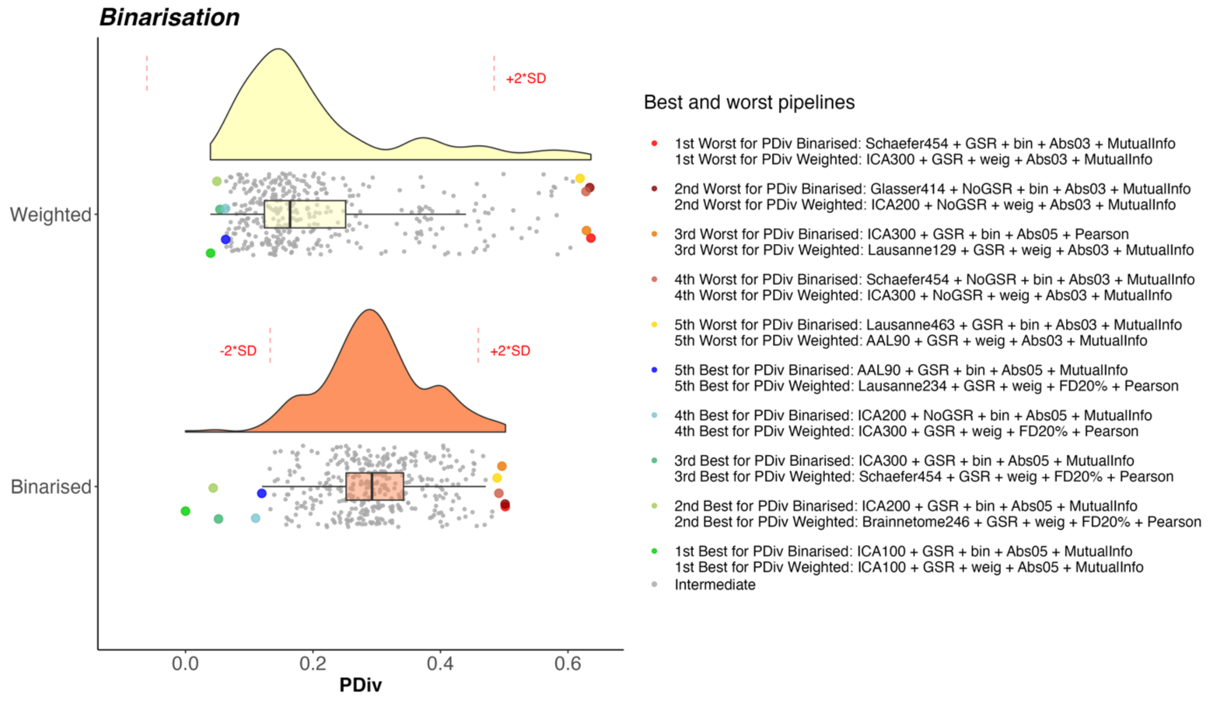

Figure S23. Portrait divergence (PDiv) by binarisation choice – NYU long-term dataset. Box-plot center line, median; box limits, upper and lower quartiles; whiskers, 1.5x interquartile range. Each data-point represents one pipeline (n=768).

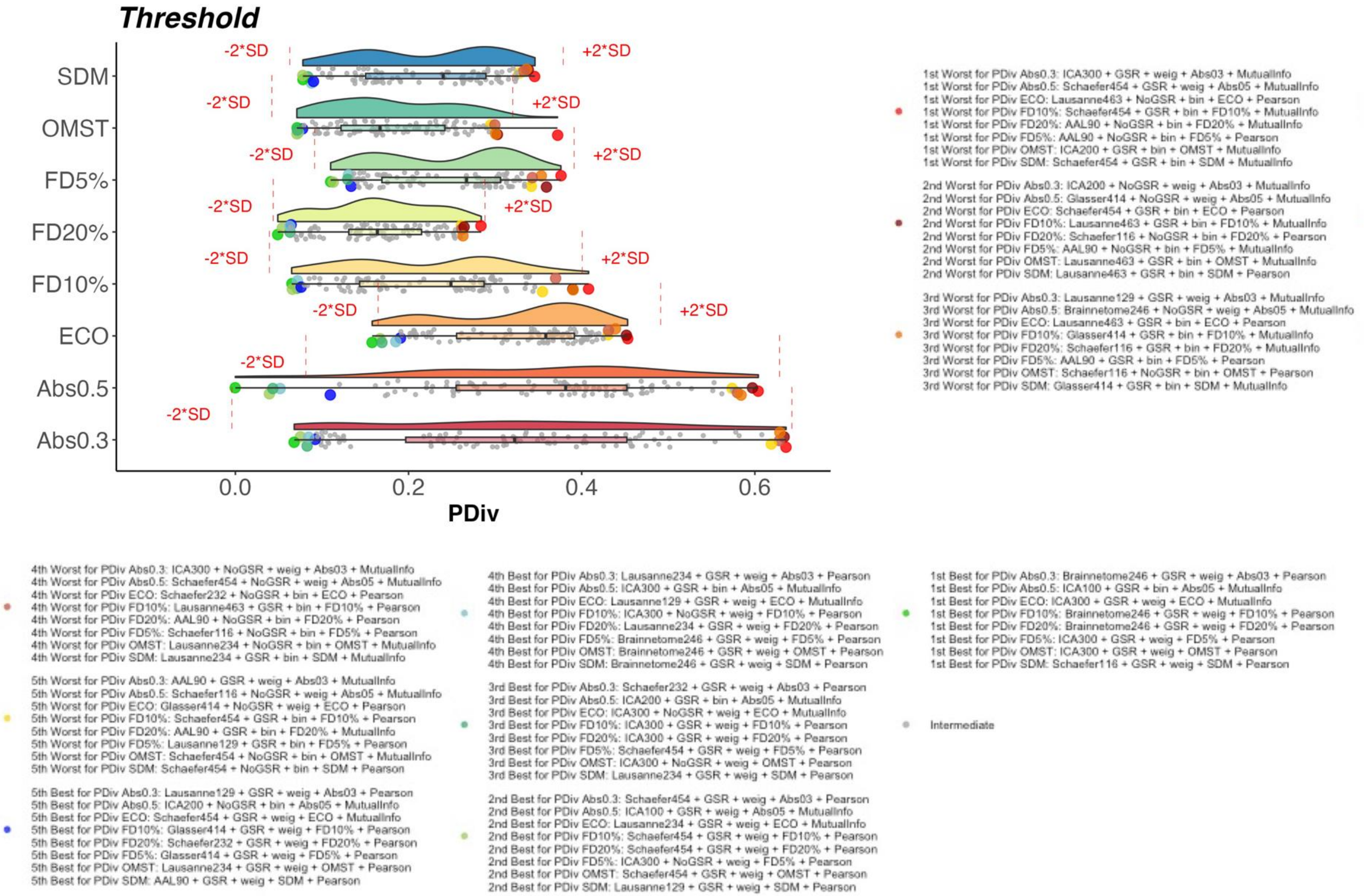

Figure S24. Portrait divergence (PDiv) by edge filtering method – NYU long-term dataset. Box-plot center line, median; box limits, upper and lower quartiles; whiskers, 1.5x interquartile range. Each data-point represents one pipeline (n=768).

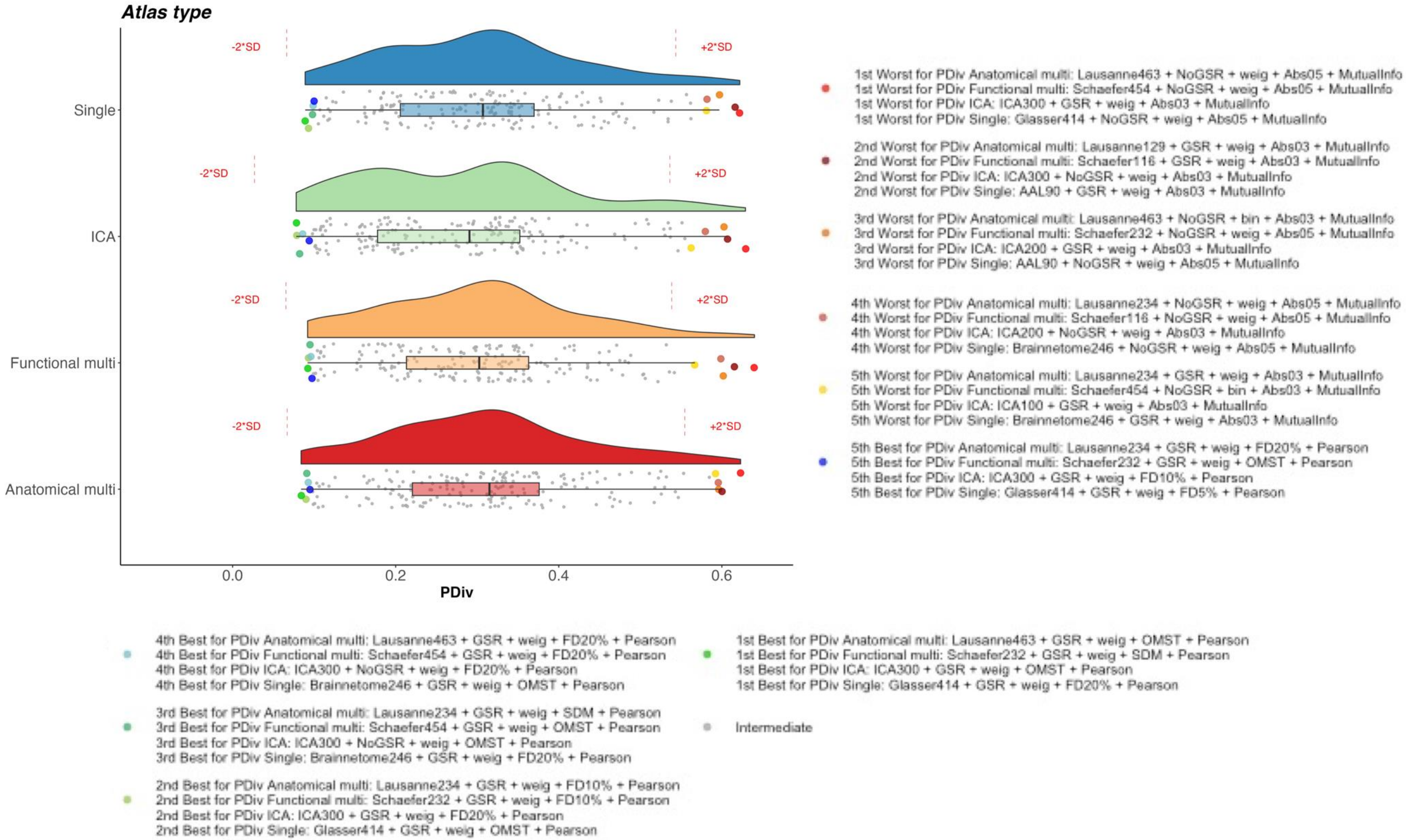

Figure S25. Portrait divergence (PDiv) by parcellation type – HCP test-retest dataset. Box-plot center line, median; box limits, upper and lower quartiles; whiskers, 1.5x interquartile range. Each data-point represents one pipeline (n=768).

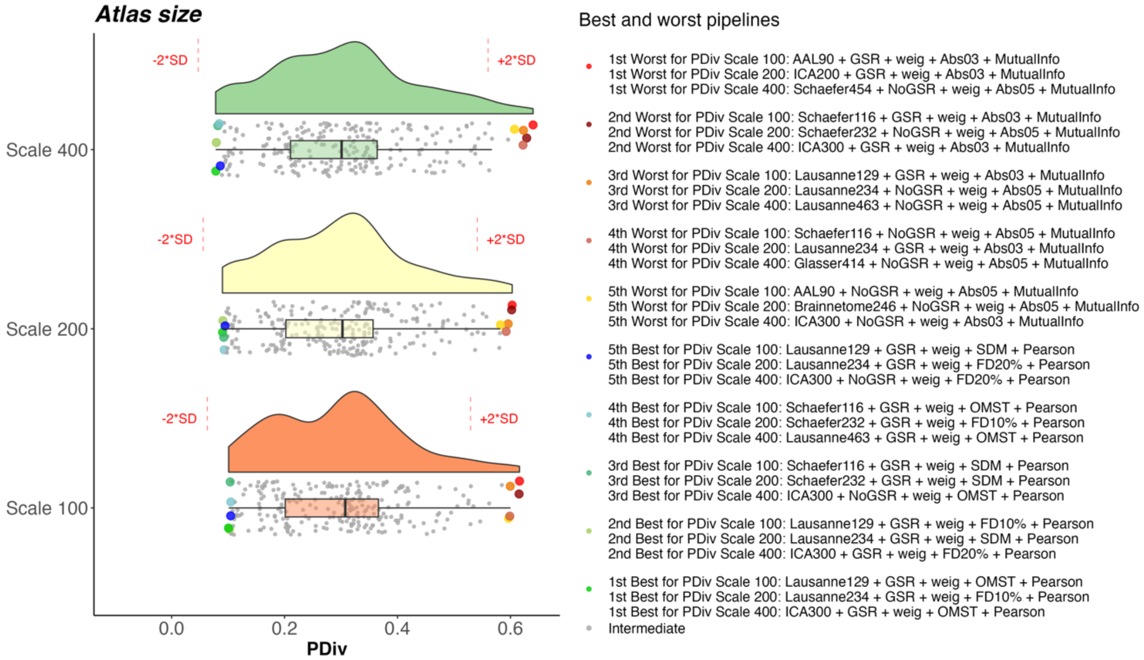

Figure S26. Portrait divergence (PDiv) by parcellation scale – HCP test-retest dataset. Box-plot center line, median; box limits, upper and lower quartiles; whiskers, 1.5x interquartile range. Each data-point represents one pipeline (n=768).

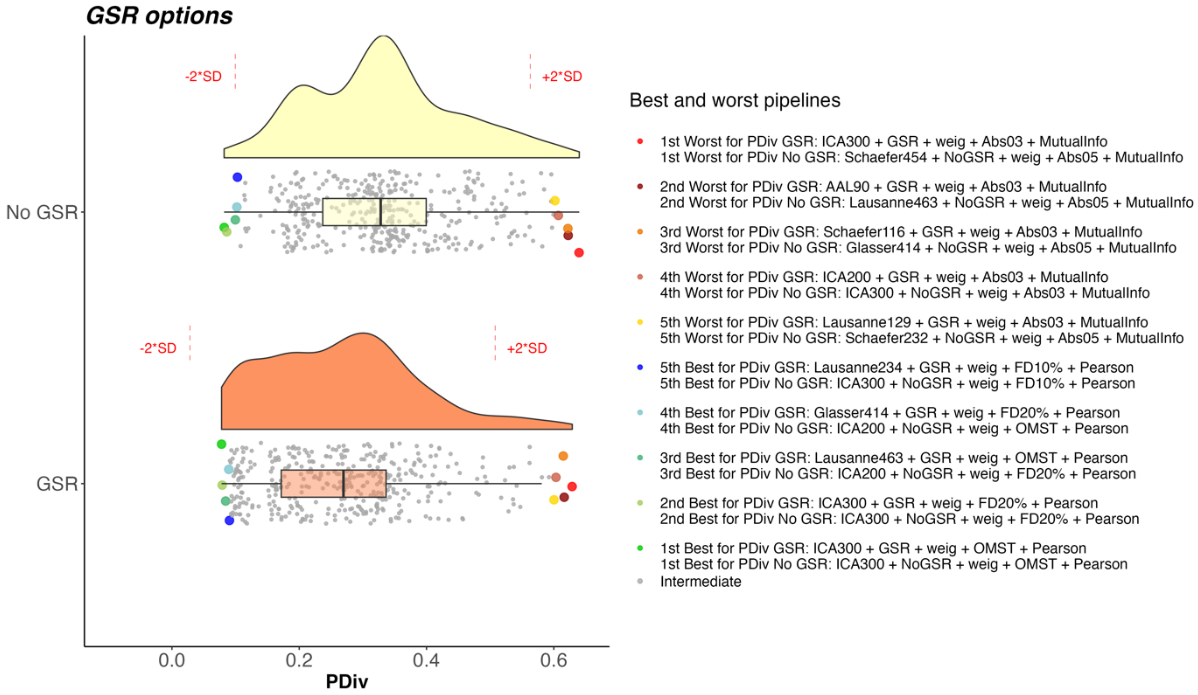

Figure S27. Portrait divergence (PDiv) by GSR use – HCP test-retest dataset. Box-plot center line, median; box limits, upper and lower quartiles; whiskers, 1.5x interquartile range. Each data-point represents one pipeline (n=768).

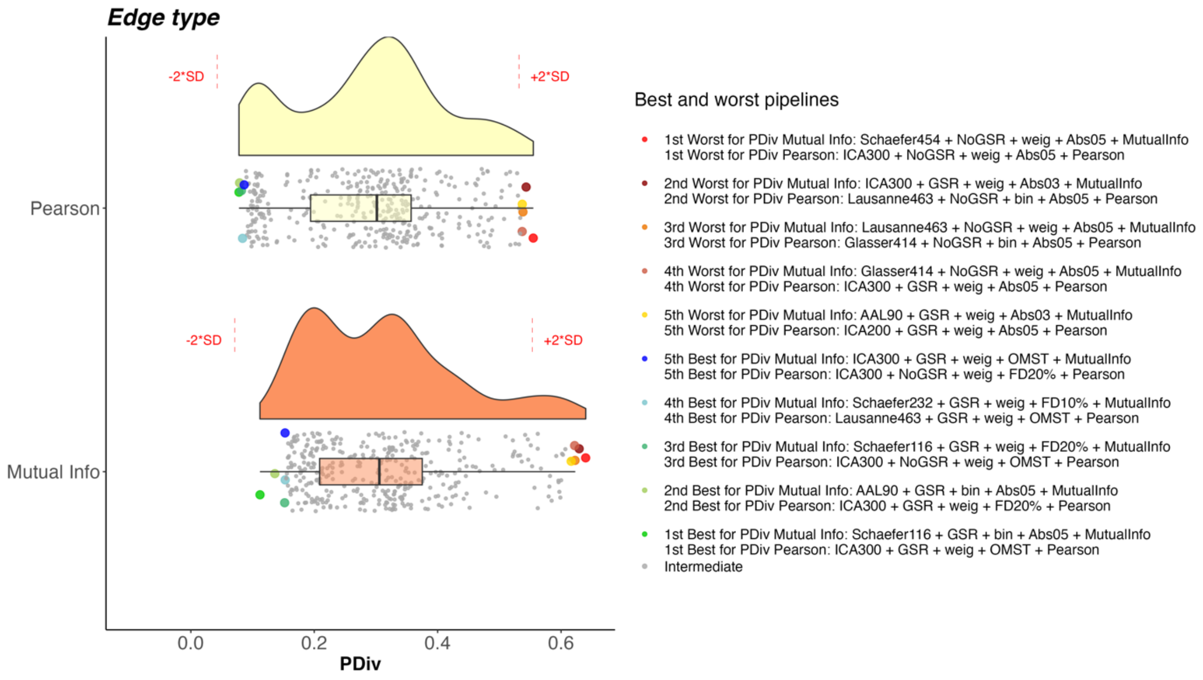

Figure S28. Portrait divergence (PDiv) by edge quantification method type – HCP test-retest dataset. Box-plot center line, median; box limits, upper and lower quartiles; whiskers, 1.5x interquartile range. Each data-point represents one pipeline (n=768).

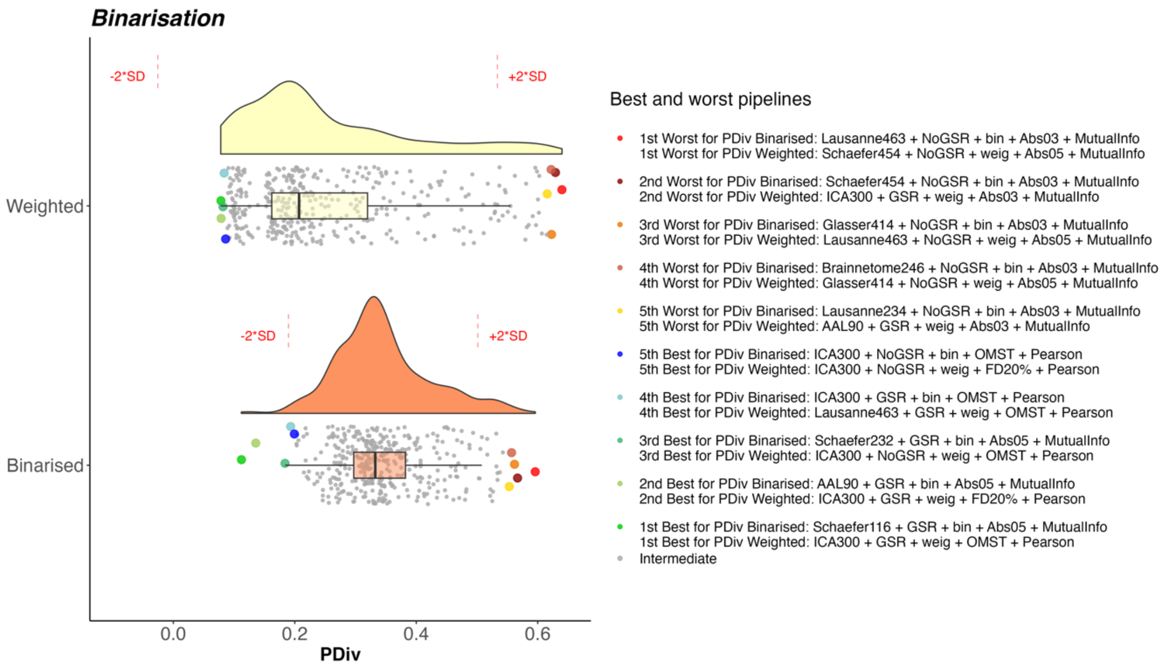

Figure S29. Portrait divergence (PDiv) by binarisation choice – HCP test-retest dataset. Box-plot center line, median; box limits, upper and lower quartiles; whiskers, 1.5x interquartile range. Each data-point represents one pipeline (n=768).

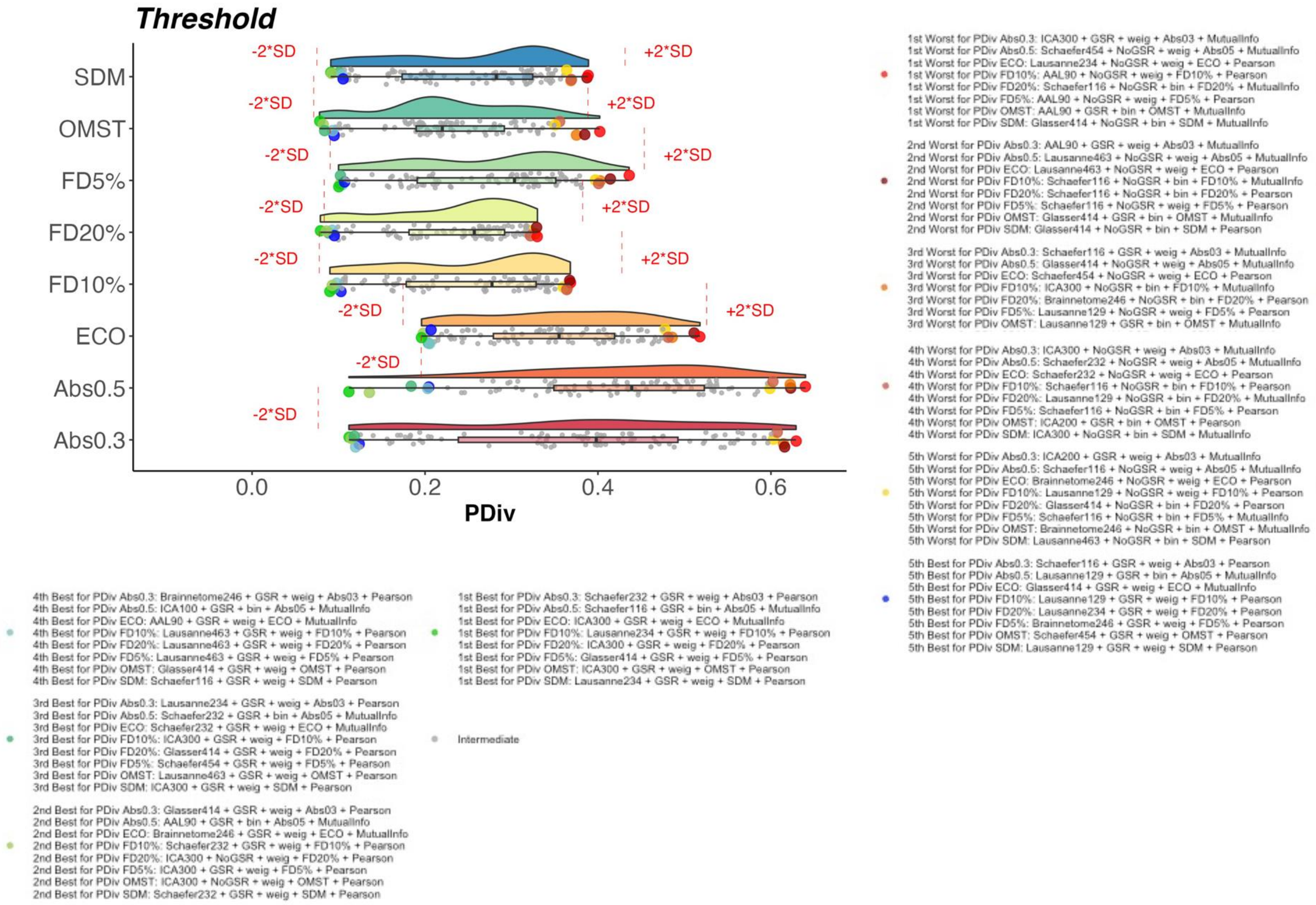

Figure S30. Portrait divergence (PDiv) by edge filtering method – HCP test-retest dataset. Box-plot center line, median; box limits, upper and lower quartiles; whiskers, 1.5x interquartile range. Each data-point represents one pipeline (n=768).

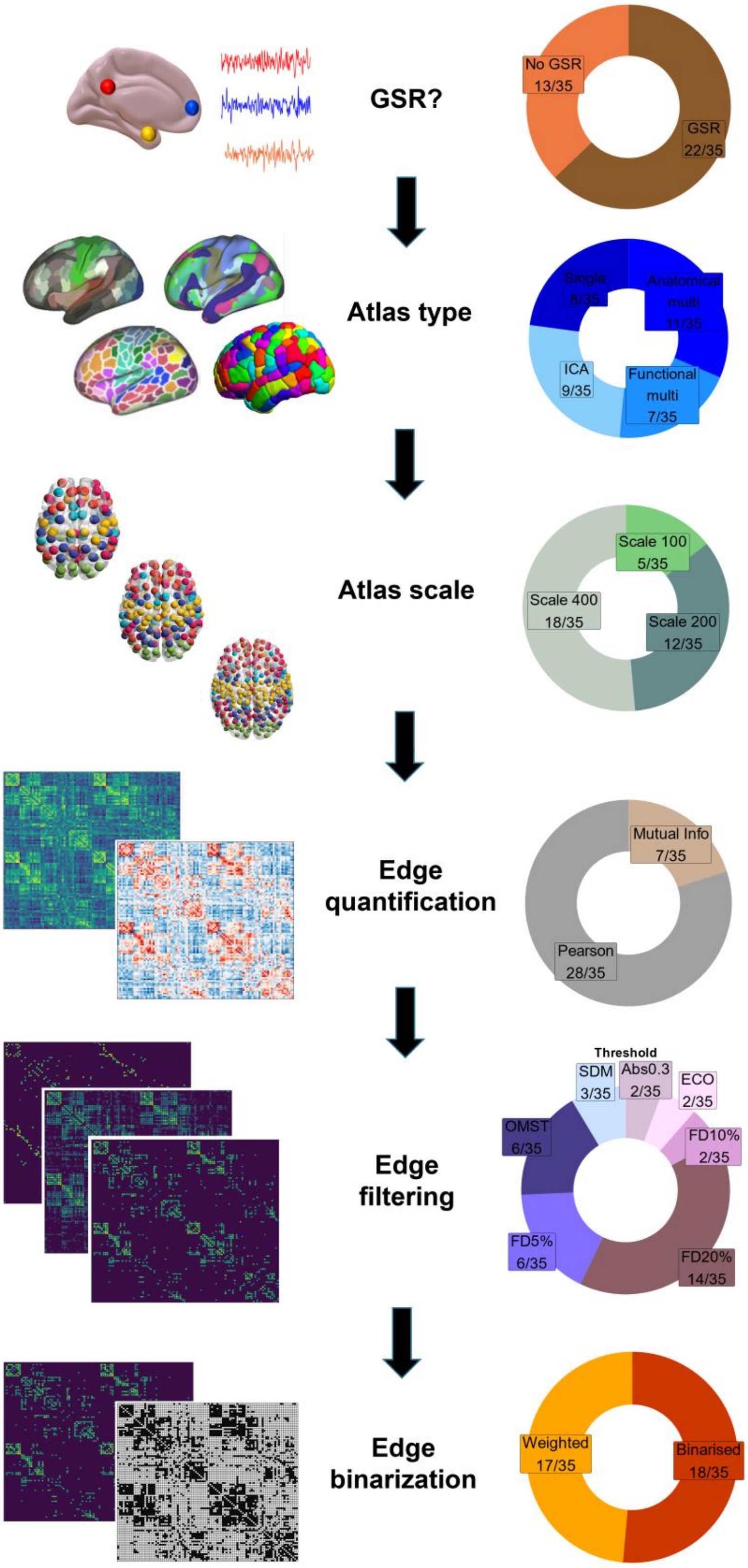

Figure S31. Prevalence of specific network construction steps among the 35 optimal pipelines, when relaxing the PDiv criterion. Pie charts demonstrate, for each network construction step, the proportion and absolute number of each option that is found among the optimal pipelines. **Abbreviations**. FD: fixed density. GSR: global signal regression. OMST: orthogonal minimal spanning tree. SDM: structural density. Illustration of parcellations adapted from Jiang et al (2023) and Zhi et al (2022).

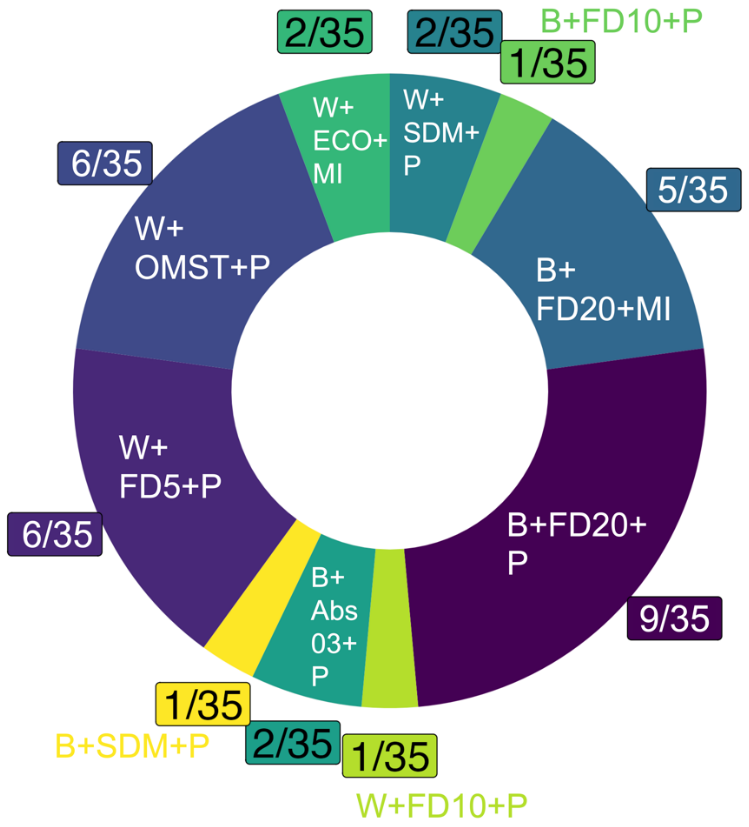

Figure S32. Optimal edge processing combinations among the top 35 pipelines, when relaxing the PDiv criterion. Pie chart displays the frequency of each combination of edge type definition, filtering, and binarisation among the 35 pipelines which fulfil all criteria for a suitable network construction pipeline. Abs03, absolute threshold (Edges with value > 0.3); B, binary edges; ECO, efficiency-cost optimisation; FD5, 5% fixed density threshold; FD10, 10% fixed density threshold; FD20, 20% fixed density threshold; OMST, orthogonal minimum spanning trees; P, Pearson correlation; MI, mutual information; SDM, structural density matching; W, weighted edges.

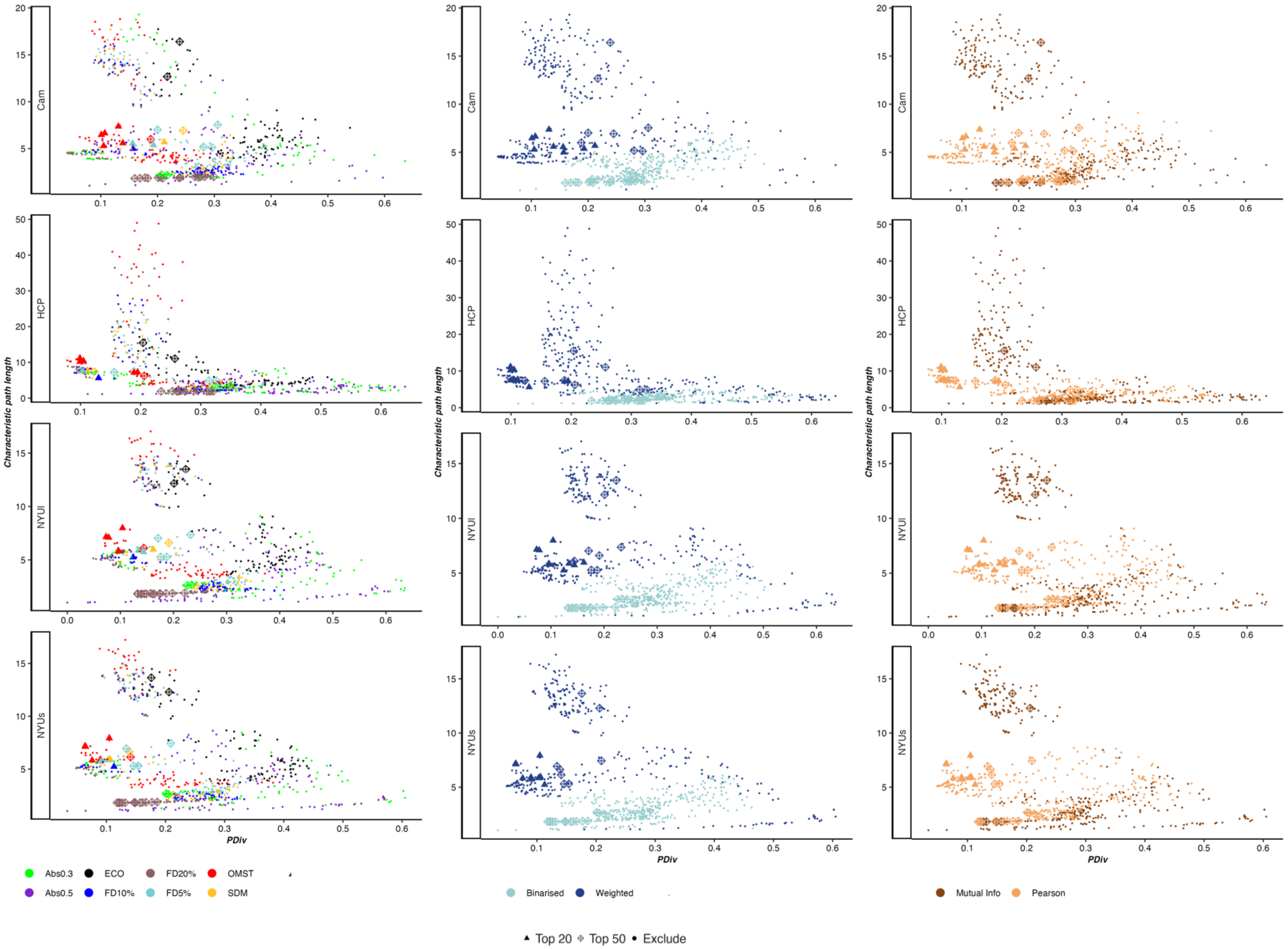

Figure S33. Test-retest PDiv versus characteristic path length of the networks produced by each pipeline (averaged across all subjects), for each dataset, as a function of filtering scheme, edge binarisation, and edge type (Pearson correlation or mutual information). Each data-point represents one pipeline; shape indicates optimality (optimal under stringent criteria, optimal under the relaxed PDiv criterion, or rejected). Each data-point represents one pipeline (n=768).

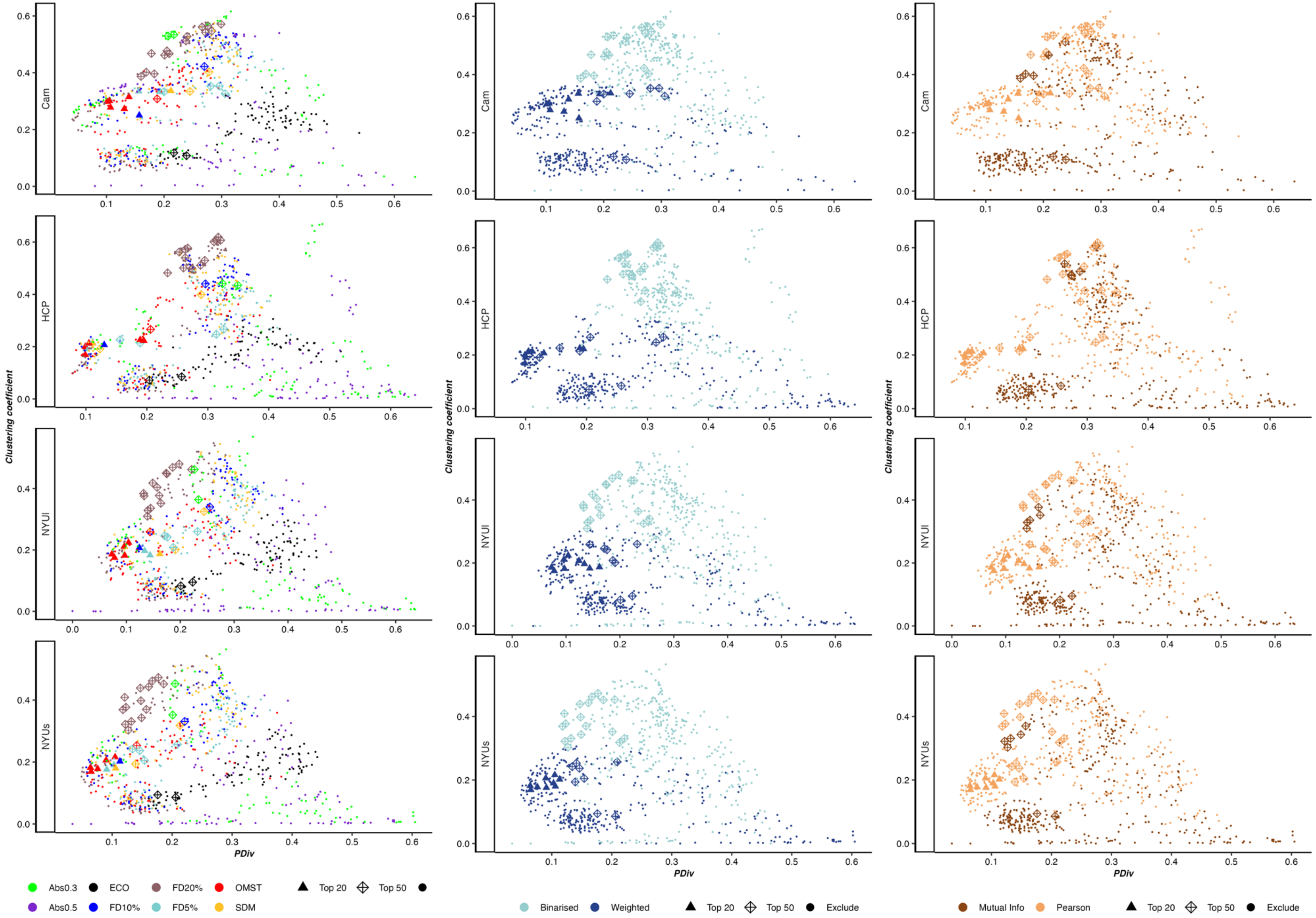

Figure S34. Test-retest PDiv versus mean clustering coefficient of the networks produced by each pipeline (averaged across all subjects), for each dataset, as a function of filtering scheme, edge binarisation, and edge type (Pearson correlation or mutual information). Each data-point represents one pipeline; shape indicates optimality (optimal under stringent criteria, optimal under the relaxed PDiv criterion, or rejected). Each data-point represents one pipeline (n=768).

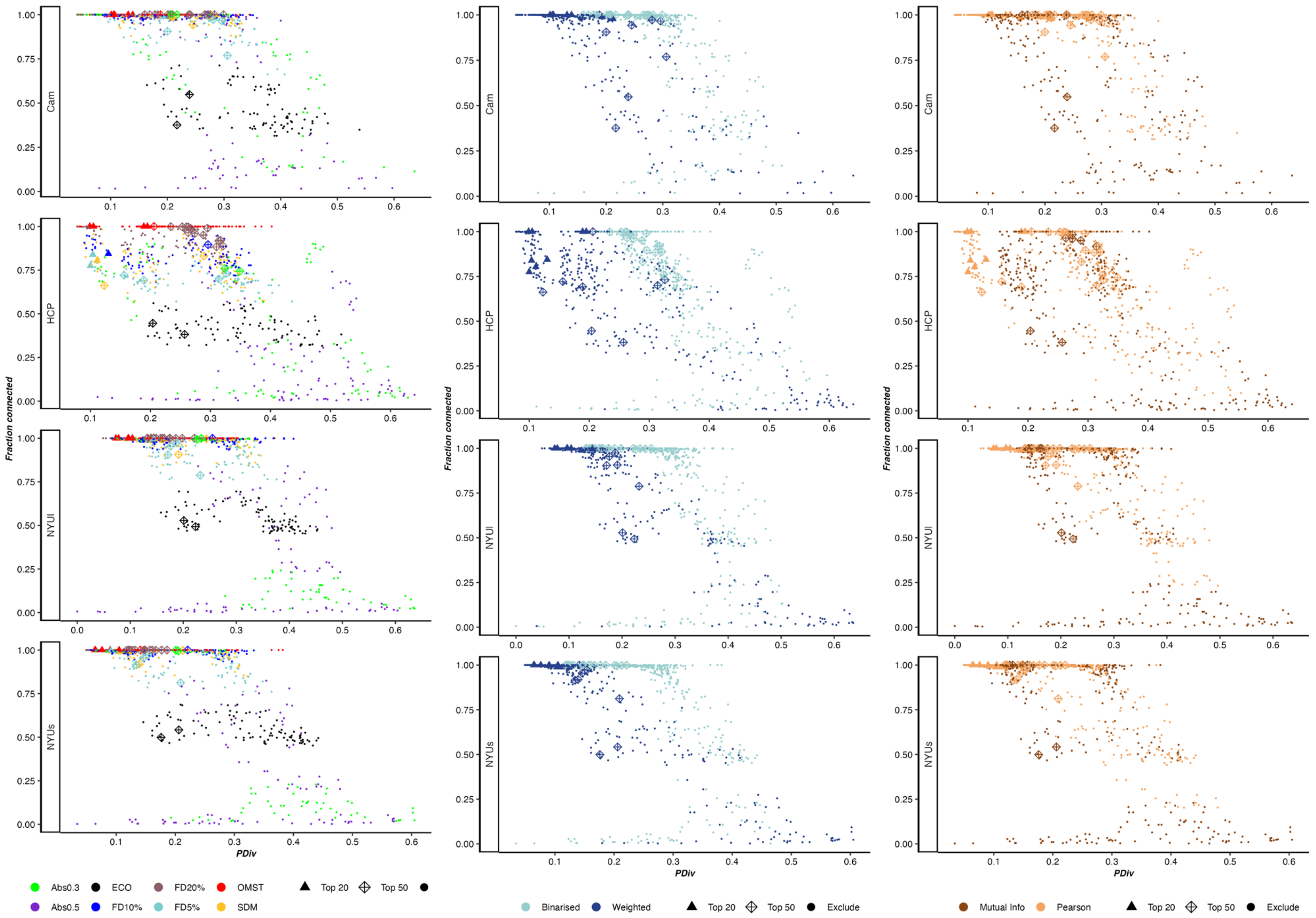

Figure S35. Test-retest PDiv versus the size of the largest connected component (as a fraction of total number of nodes) of the networks produced by each pipeline (averaged across all subjects), for each dataset, as a function of filtering scheme, edge binarisation, and edge type (Pearson correlation or mutual information). Each data-point represents one pipeline; shape indicates optimality (optimal under stringent criteria, optimal under the relaxed PDiv criterion, or rejected). Each data-point represents one pipeline (n=768).

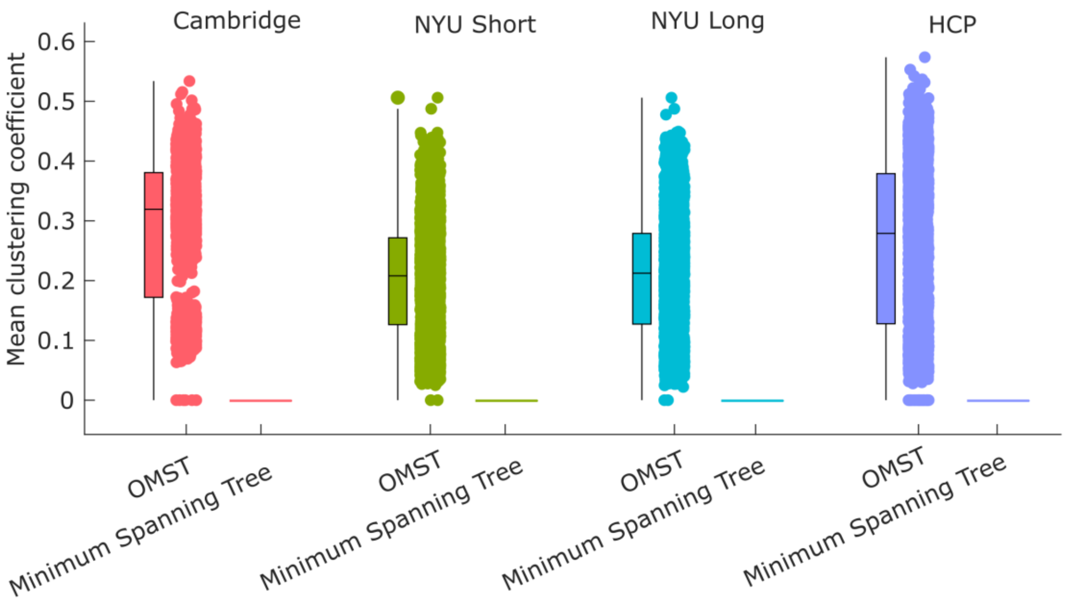

Figure S36. Comparing mean clustering coefficient of the network, for OMST and Minimum Spanning Tree filtering schemes. Separately for each test-retest datasets, each data-point represents an individual functional connectome, reconstructed with one of the n=72 pipelines that use OMST filtering, or an equivalent pipeline but using Minimum Spanning Tree filtering instead. The mean clustering coefficient is always zero when using Minimum Spanning Tree filtering, regardless of any other network construction choice. Box-plot center line, median; box limits, upper and lower quartiles; whiskers, 1.5x interquartile range.

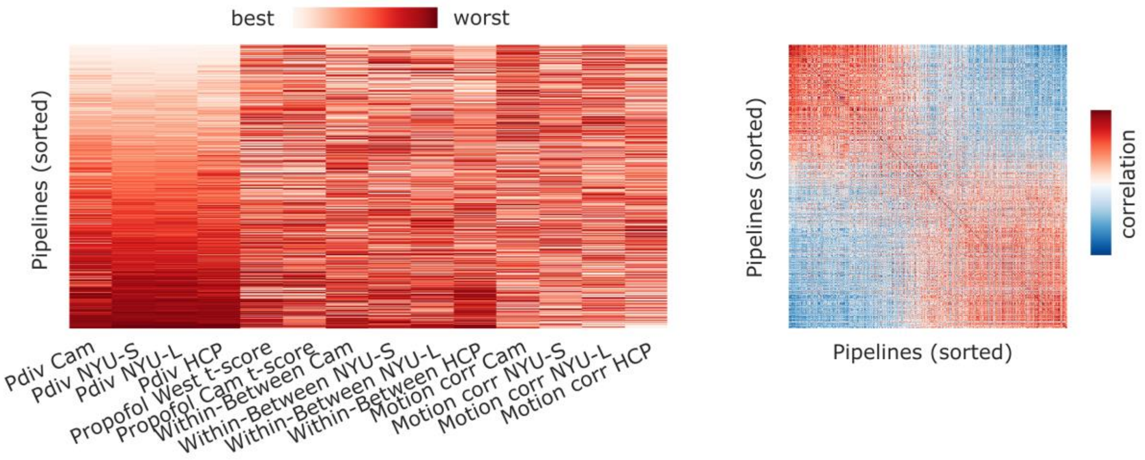

Figure S37. Pipelines' ranked performance for each criterion and dataset, sorted by overall rank. Left: For the Pdiv criterion, best performance refers to the smallest PDiv; for the propofol criterion, best performance is the greatest t-score in the correct direction; for the within-between criterion, best performance means the greatest proportion of participants for whom the within-subjects Pdiv is smaller than between-subjects Pdiv; for the motion correlation criterion, best performance is identified as the smallest magnitude of correlation with motion. The empty networks criterion is not included, since it is binary. Overall rank is the mean across all columns. Right: correlation between each pair of pipelines in terms of performance, sorted by overall rank.

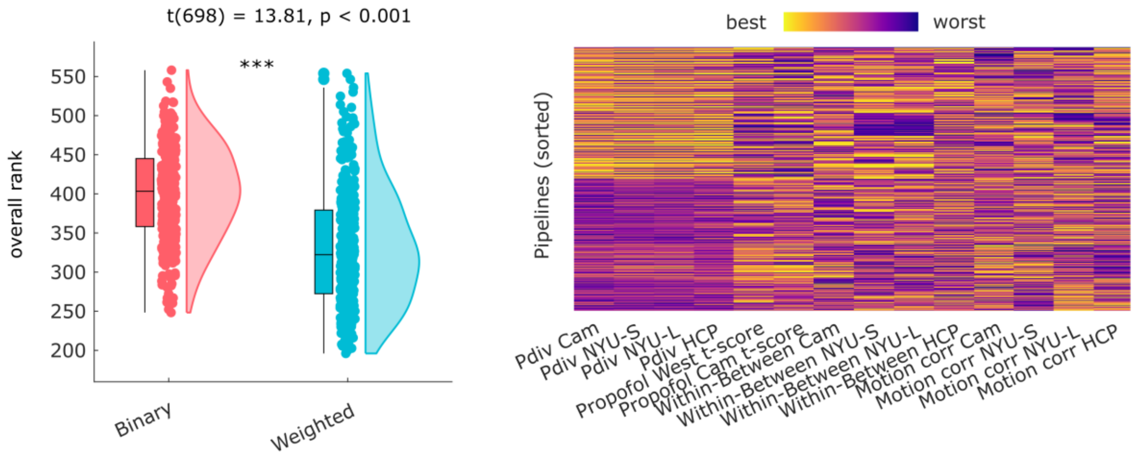

Figure S38. Statistical comparison of pipelines' performance as a function of binarization choice. Performance is quantified as overall rank (mean rank across all criteria and datasets, with lower rank indicating better performance). Top: Each data-point indicates one pipeline. The empty-networks criterion was not included: instead, pipelines failing this criterion were excluded from the analysis. Box-plots indicate the median and inter-quartile range of each distribution. Bottom: ranked performance across all datasets and criteria, with pipelines sorted by binarization choice. Box-plot center line, median; box limits, upper and lower quartiles; whiskers, 1.5x interquartile range. ***, $p < 0.001$ from independent samples t-test (two-sided).

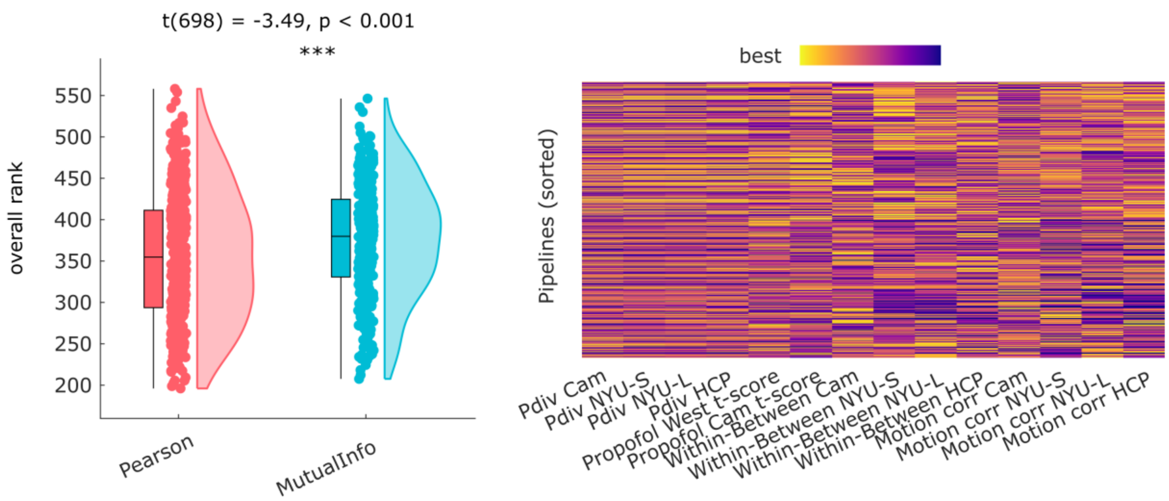

Figure S39. Statistical comparison of pipelines' performance as a function of edge type. Performance is quantified as overall rank (mean rank across all criteria and datasets, with lower rank indicating better performance). Top: Each data-point indicates one pipeline. The empty-networks criterion was not included: instead, pipelines failing this criterion were excluded from the analysis. Box-plots indicate the median and inter-quartile range of each distribution. Bottom: ranked performance across all datasets and criteria, with pipelines sorted by edge type. Box-plot center line, median; box limits, upper and lower quartiles; whiskers, 1.5x interquartile range. ***, $p < 0.001$ from independent samples t-test (two-sided).

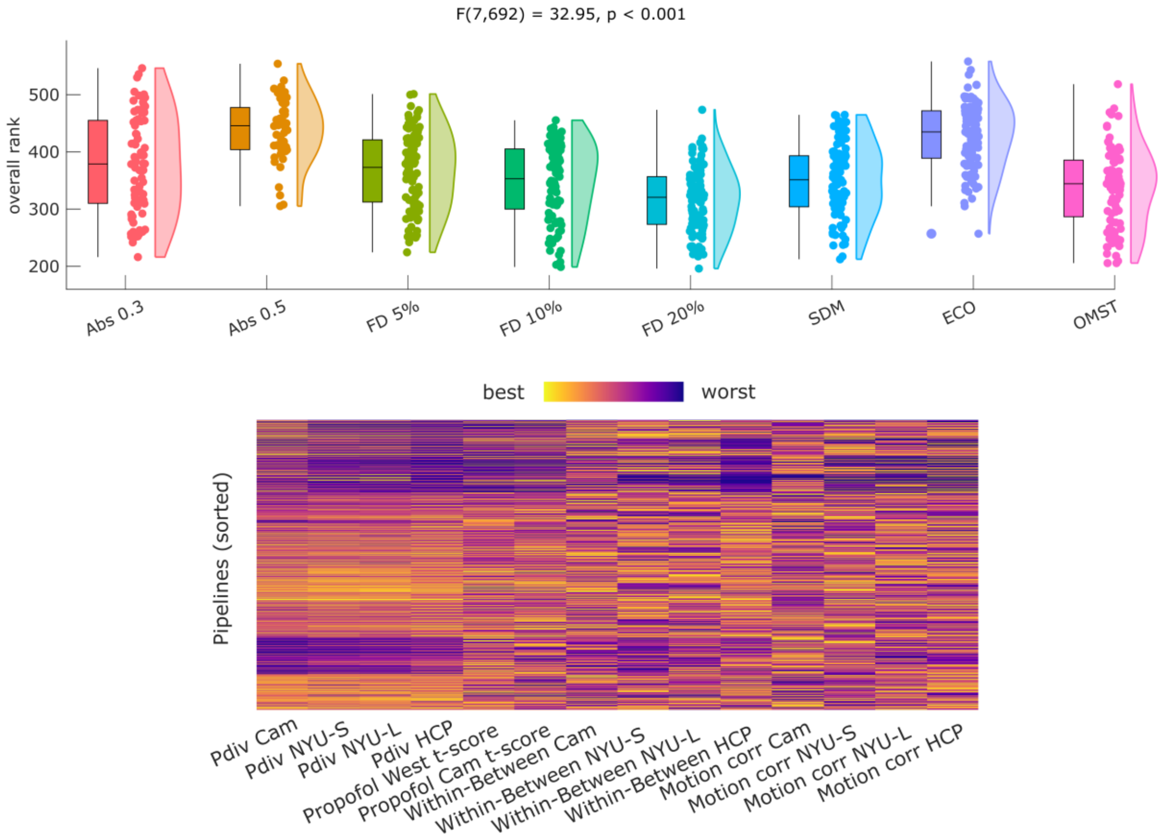

Figure S40. Statistical comparison of pipelines' performance as a function of filtering scheme. Performance is quantified as overall rank (mean rank across all criteria and datasets, with lower rank indicating better performance). Top: Each data-point indicates one pipeline. The empty-networks criterion was not included: instead, pipelines failing this criterion were excluded from the analysis. Box-plots indicate the median and inter-quartile range of each distribution. Bottom: ranked performance across all datasets and criteria, with pipelines sorted by filtering scheme. Box-plot center line, median; box limits, upper and lower quartiles; whiskers, 1.5x interquartile range.

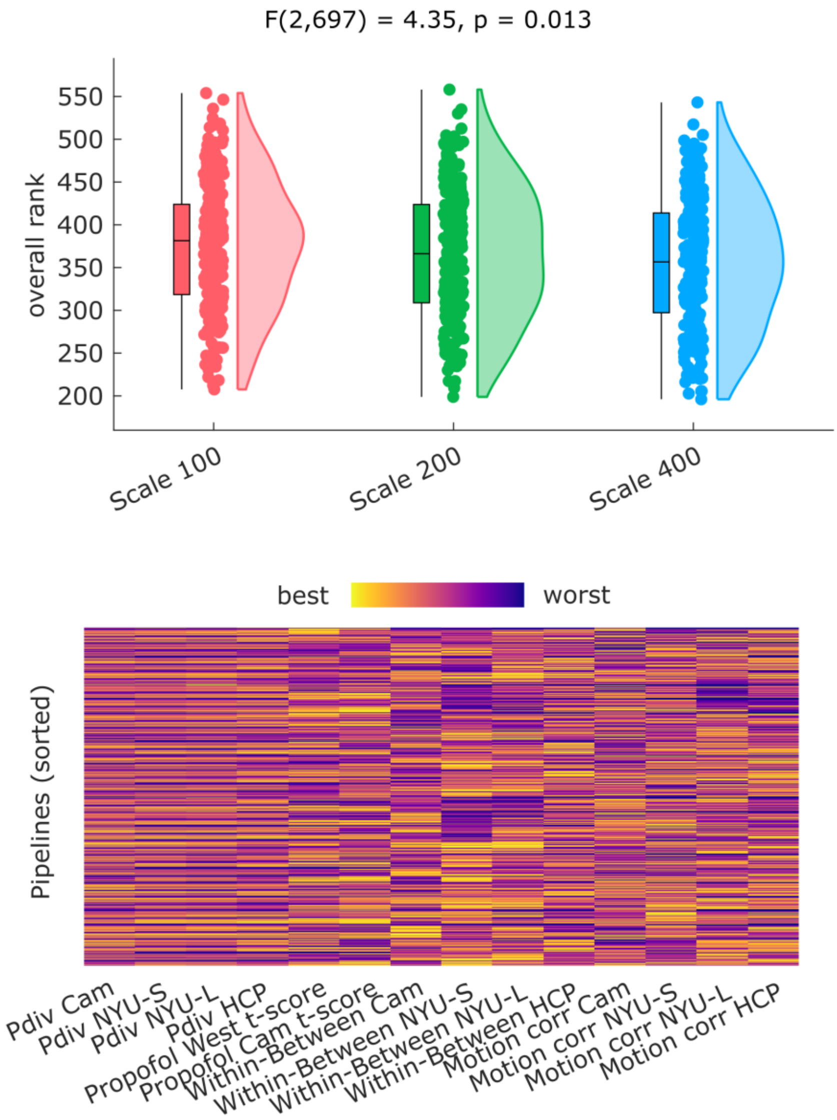

Figure S41. Statistical comparison of pipelines' performance as a function of parcellation scale. Performance is quantified as overall rank (mean rank across all criteria and datasets, with lower rank indicating better performance). Top: Each data-point indicates one pipeline. The empty-networks criterion was not included: instead, pipelines failing this criterion were excluded from the analysis. Box-plots indicate the median and inter-quartile range of each distribution. Bottom: ranked performance across all datasets and criteria, with pipelines sorted by parcellation scale. Box-plot center line, median; box limits, upper and lower quartiles; whiskers, 1.5x interquartile range.

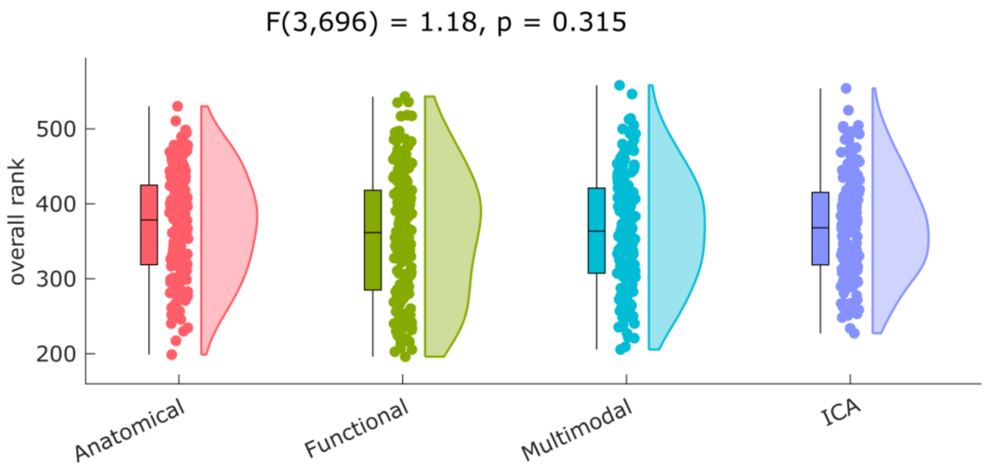

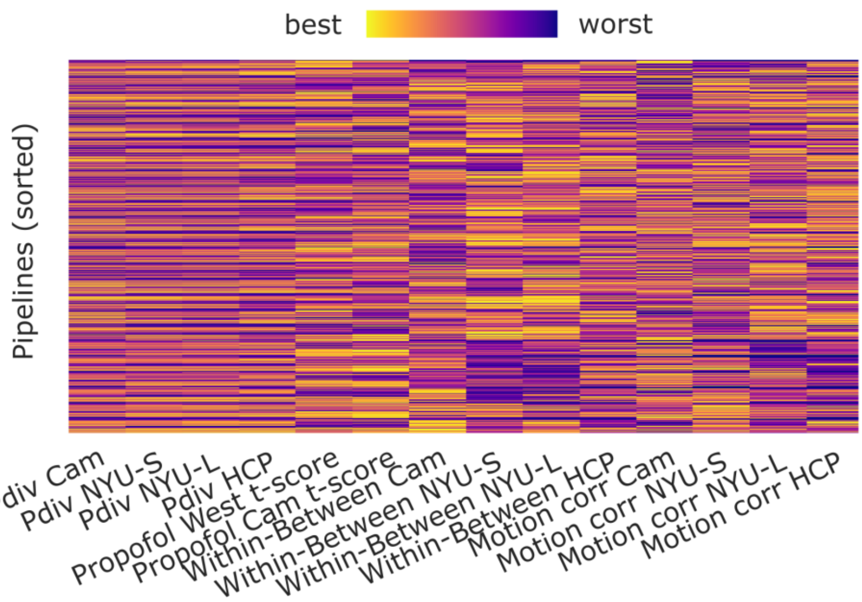

Figure S42. Statistical comparison of pipelines' performance as a function of parcellation type. Performance is quantified as overall rank (mean rank across all criteria and datasets, with lower rank indicating better performance). Top: Each data-point indicates one pipeline. The empty-networks criterion was not included: instead, pipelines failing this criterion were excluded from the analysis. Box-plots indicate the median and inter-quartile range of each distribution. Bottom: ranked performance across all datasets and criteria, with pipelines sorted by parcellation type. Box-plot center line, median; box limits, upper and lower quartiles; whiskers, 1.5x interquartile range.

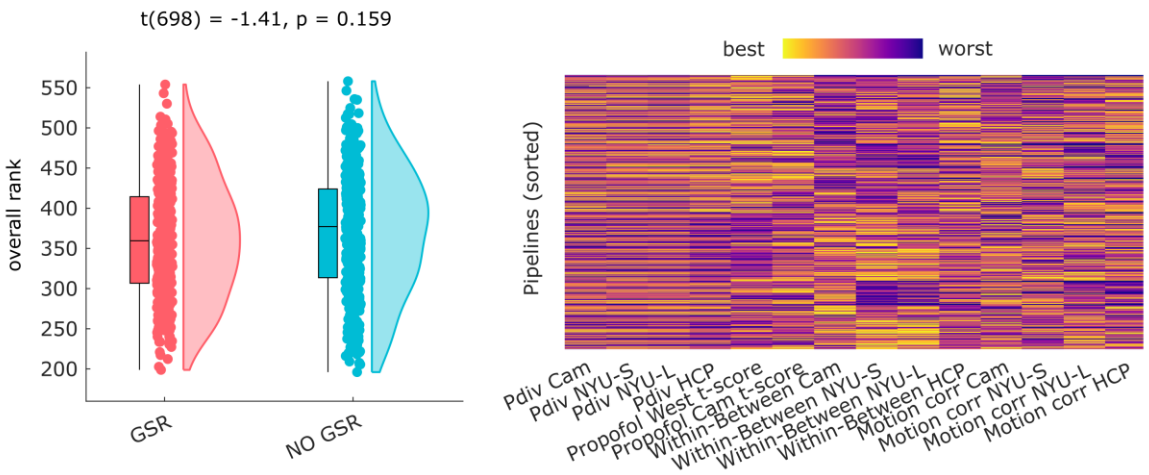

Figure S43. Statistical comparison of pipelines' performance as a function of GSR use. Performance is quantified as overall rank (mean rank across all criteria and datasets, with lower rank indicating better performance). Top: Each data-point indicates one pipeline. The empty-networks criterion was not included: instead, pipelines failing this criterion were excluded from the analysis. Box-plots indicate the median and inter-quartile range of each distribution. Bottom: ranked performance across all datasets and criteria, with pipelines sorted by GSR use. Box-plot center line, median; box limits, upper and lower quartiles; whiskers, 1.5x interquartile range.
