# Supplementary material for: Systematic evaluation of fMRI data-processing pipelines for consistent functional connectomics
Source: Nat Commun. 2024 Jun 4;15:4745. doi: 10.1038/s41467-024-48781-5 (PMC11150439; doi:10.1038/s41467-024-48781-5)
Supplement: Supplementary file 2 — Description of Additional Supplementary Files [file 41467_2024_48781_MOESM2_ESM.pdf]

Description of Additional Supplementary Files:

Supplementary Data 1: Final selection of pipelines that meet all criteria.

Supplementary Data 2: Interactive Pipeline Selection Tool, providing a breakdown of each pipeline's performance across all criteria and all datasets.
