# Supplementary material for: Systematic evaluation of fMRI data-processing pipelines for consistent functional connectomics
Source: Nat Commun. 2024 Jun 4;15:4745. doi: 10.1038/s41467-024-48781-5 (PMC11150439; doi:10.1038/s41467-024-48781-5)
Supplement: Supplementary file 5 — Reporting Summary [file 41467_2024_48781_MOESM5_ESM.pdf]

nature portfolio

Corresponding author(s): Andrea Luppi

Last updated by author(s): Mar 19, 2024

Reporting Summary

Nature Portfolio wishes to improve the reproducibility of the work that we publish. This form provides structure for consistency and transparency in reporting. For further information on Nature Portfolio policies, see our Editorial Policies and the Editorial Policy Checklist.

Statistics

For all statistical analyses, confirm that the following items are present in the figure legend, table legend, main text, or Methods section.

n/a	Confirmed	
☐	☒	The exact sample size (*n*) for each experimental group/condition, given as a discrete number and unit of measurement
☐	☒	A statement on whether measurements were taken from distinct samples or whether the same sample was measured repeatedly
☐	☒	The statistical test(s) used AND whether they are one- or two-sided<br>*Only common tests should be described solely by name; describe more complex techniques in the Methods section.*
☒	☐	A description of all covariates tested
☒	☐	A description of any assumptions or corrections, such as tests of normality and adjustment for multiple comparisons
☐	☒	A full description of the statistical parameters including central tendency (e.g. means) or other basic estimates (e.g. regression coefficient) AND variation (e.g. standard deviation) or associated estimates of uncertainty (e.g. confidence intervals)
☐	☒	For null hypothesis testing, the test statistic (e.g. *F*, *t*, *r*) with confidence intervals, effect sizes, degrees of freedom and *P* value noted<br>*Give P values as exact values whenever suitable.*
☒	☐	For Bayesian analysis, information on the choice of priors and Markov chain Monte Carlo settings
☒	☐	For hierarchical and complex designs, identification of the appropriate level for tests and full reporting of outcomes
☐	☒	Estimates of effect sizes (e.g. Cohen's *d*, Pearson's *r*), indicating how they were calculated

Our web collection on statistics for biologists contains articles on many of the points above.

Software and code

Policy information about availability of computer code

Data collection	The TIVA Trainer (European Society for Intravenous Aneaesthesia, eurosiva.eu) pharmacokinetic simulation program was used to control the propofol infusion. An Alaris PK infusion pump (Carefusion, Basingstoke, UK) was used for propofol infusion.
Data analysis	The CONN toolbox is freely available online (http://www.nitrc.org/projects/conn). Python (v3.6) code for the portrait divergence is freely available online (https://github.com/bagrow/network-portrait-divergence). MATLAB (v2019a) code for the Orthogonal Minimum Spanning Tree thresholding is freely available online (https://github.com/stdimitr/topological_filtering_networks). The Brain Connectivity Toolbox code used for graph-theoretical analyses is freely available online (https://sites.google.com/site/bctnet/). FSL (FMRIB Software Library) v6 toolbox is freely available for academic research at https://fsl.fmrib.ox.ac.uk/.

For manuscripts utilizing custom algorithms or software that are central to the research but not yet described in published literature, software must be made available to editors and reviewers. We strongly encourage code deposition in a community repository (e.g. GitHub). See the Nature Portfolio guidelines for submitting code & software for further information.

Data

Policy information about availability of data

All manuscripts must include a data availability statement. This statement should provide the following information, where applicable:

- Accession codes, unique identifiers, or web links for publicly available datasets
- A description of any restrictions on data availability
- For clinical datasets or third party data, please ensure that the statement adheres to our policy

Source data are provided with this paper. The NYU dataset is freely available from the International Neuroimaging Data-Sharing Initiative (INDI) (http://www.nitrc.org/projects/nyu_trt). The Cambridge datasets are available upon request from author EAS (email: eas46@cam.ac.uk). The Western propofol dataset is available on the OpenNeuro data repository (doi: 10.18112/openneuro.ds003171.v2.0.1). The HCP data are available from https://www.humanconnectome.org/. The AAL atlas is available online at https://www.gin.cnrs.fr/en/tools/aal/. The Brainnetome atlas is available online at https://atlas.brainnetome.org/download.html. The Glasser parcellation is available online at https://balsa.wustl.edu/study/show/RVVG. The Lausanne multi-scale atlas can be obtained from https://github.com/mattcieslak/easy_lausanne. The Schaefer multi-scale atlas is available at https://github.com/ThomasYeoLab/CBIG/tree/master/stable_projects/brain_parcellation/Schaefer2018_LocalGlobal. The Tian subcortical multi-scale atlas is available at https://github.com/yetianmed/subcortex. The Group-ICA parcellations are available from https://www.humanconnectome.org/.

Human research participants

Policy information about studies involving human research participants and Sex and Gender in Research.

Reporting on sex and gender	Each dataset had been previously collected, and included subjects of both sexes. The design is within-subjects in each dataset, and our focus was not on comparing groups or inter-individual differences but rather on comparing pipelines.
Population characteristics	See Behavioural & Social sciences reporting.
Recruitment	NYU Test-Retest dataset: see original publication.<br><br>HCP dataset: Detailed information about the recruitment, acquisition and imaging is provided in the dedicated HCP publications.<br><br>Cambridge Test-Retest dataset: advertisements in the Cambridge area. Exclusion criteria included National Adult Reading Test (NART) <70, Mini Mental State Examination (MMSE) <23, left- handedness, history of drug/alcohol abuse, history of psychiatric or neurological disorders, contraindications for MRI scanning, medication that may affect cognitive performance or prescribed for depression, and any physical handicap that could prevent the completion of testing.<br><br>Cambridge propofol dataset: healthy volunteers were recruited via advertisements placed throughout central Cambridge, UK. Exclusion criteria included history of psychiatric or neurological disorders, or contraindications to MRI scanning.<br><br>Western propofol dataset: participants were recruited with posters around campus as per ethics protocol. Participants approaches the research team to seek participation, and there were no specific selection biases. Participants were required to be healthy, right-handed, native English speakers with no history of neurological disorders, and no contraindications to MRI scanning.
Ethics oversight	NYU Test-Retest dataset: The study was approved by the institutional review boards of the New York University School of Medicine and New York University, and participants provided written informed consent and were compensated for their participation.<br>All HCP scanning protocols were approved by the local Institutional Review Board at Washington University in St. Louis.<br>Cambridge Test-Retest dataset: Cambridgeshire 2 Research Ethics Committee approved the study (LREC 08/H0308/246) and all volunteers gave written informed consent before participating.<br>Cambridge propofol dataset: Ethical approval for these studies was obtained from the Cambridgeshire 2 Regional Ethics Committee, and all subjects gave informed consent to participate in the study.<br>Western propofol dataset: The study received ethical approval from the Health Sciences Research Ethics Board and Psychology Research Ethics Board of Western University (Ontario, Canada). In accordance with relevant ethical guidelines, each volunteer provided written informed consent, and received monetary compensation for their time.

Note that full information on the approval of the study protocol must also be provided in the manuscript.

Field-specific reporting

Please select the one below that is the best fit for your research. If you are not sure, read the appropriate sections before making your selection.

☐ Life sciences ☒ Behavioural & social sciences ☐ Ecological, evolutionary & environmental sciences

For a reference copy of the document with all sections, see nature.com/documents/nr-reporting-summary-flat.pdf

Behavioural & social sciences study design

All studies must disclose on these points even when the disclosure is negative.

Study description

This study uses quantitative methods. We processed the same data using different data processing pipelines,a nd evaluated the resulting functional brain networks across a battery of criteria.

Research sample

We used pre-existing datasets; no new data were collected for this study. The sample sizes were determined by the original investigators of each study and are in line with the published literature.

NYU Test-Retest dataset: 25 participants (mean age 30.7 ± 8.8 years, 10 males) with no history of psychiatric or neurological illness.
HCP Test-retest dataset: the 1200 Human Connectome Project subjects include a subset of 46 healthy individuals (13 male, age 22–35 years), who were each scanned twice at 3T, at intervals ranging between 1 month and 11 months)
Cambridge Test-Retest dataset: Right-handed healthy participants (N=22, age range, 19–57 years; mean age, 35.0 years; SD 11.2; female-to-male ratio, 9/13) were recruited via advertisements in the Cambridge area and were paid for their participation.
Cambridge propofol dataset: 16 healthy volunteer subjects were initially recruited for scanning. In addition to the original 16 volunteers, data were acquired for nine participants using the same procedures, bringing the total number of participants in this dataset to 25 (11 males, 14 females; mean age 34.7 years, SD = 9.0 years).
Western propofol dataset: Healthy volunteers (n=19) were recruited (18–40 years; 13 males). Volunteers were right-handed, native English speakers, and had no history of neurological disorders.

Sampling strategy

No power analysis was performed prior to data collection, since we did not collect these data ourselves for this study, but rather used existing data; the sample sizes are within the range reported in the literature. Each study recruited healthy volunteers according to the respective criteria of the original investigators (see Recruitment above).

Data collection

NYU Test-Retest dataset: no experimental manipulation was present for the purposes of the present resting-state analysis.

HCP dataset: no experimental manipulation was present for the purposes of the present resting-state analysis.

Cambridge Test-Retest dataset: no experimental manipulation was present for the purposes of the present resting-state analysis.

Cambridge propofol dataset: Two senior anaesthetists were present during scanning sessions and observed the subjects throughout the study from the MRI control room and on a video link that showed the subject in the scanner. They could not be blinded to experimental condition, since part of their role involved determining the participants' level of anaesthesia. Electrocardiography and pulse oximetry were performed continuously, and measurements of heart rate, non-invasive blood pressure, and oxygen saturation were recorded at regular intervals. Propofol was administered intravenously as a “target controlled infusion” (plasma concentration mode), using an Alaris PK infusion pump (Carefusion, Basingstoke, UK). Blood samples were drawn towards the end of each titration period and before the plasma target was altered, to assess plasma propofol levels.

Western propofol dataset: two senior anaesthesiologists and one anaesthesia nurse were present, and they could not be blinded to experimental condition, since part of their role involved determining the participants' level of anaesthesia. Propofol was administered intravenously using an Baxter AS50 auto syringe infusion pump (Baxter Healthcare, Singapore).

Timing

NYU Test-Retest dataset: Scans 2 and 3 were conducted in a single scan session, 45 min apart, which took place on average 11 months (range 5–16 months) after Scan 1.
HCP Test-retest dataset: participants were scanned twice, 1-11 months apart.
Cambridge Test-Retest dataset: data were collected between October 2009 and September 2010, with the second session taking place 2–4 weeks after the first.
Cambridge propofol dataset: the first 16 volunteers were scanned between June and October 2008; additional 9 volunteers were scanned between March 2009 and November 2011;
Western propofol dataset: data were collected between May and November 2014.

Data exclusions

NYU Test-Retest dataset: all 25 available subjects were included.
HCP Test-Retest dataset: all 46 available subjects were included.
Cambridge Test-Retest dataset: participants could only be included if they had usable data for both scanning sessions. This left N=18 participants.
Cambridge propofol dataset: 10 participants were excluded, either because of incomplete scans (n=2), or due of excessive motion in the scanner (n=8, 5mm maximum motion threshold).
Western propofol dataset: Due to equipment malfunction or physiological impediments to anaesthesia in the scanner, data from three healthy volunteers (1 male) from the propofol dataset were excluded from analyses, leaving 16 volunteers for analysis.
All exclusions took place before the data were analysed.

Non-participation

No participants declined participation.

Randomization

For the NYU Test-Retest dataset, Cambridge Test-retest dataset, and HCP test-retest dataset, no randomisation was present, since there were no effects of interest beyond the passage of time: each individual was scanned at each time-point, ie both at test and at

re-test, so this was a within-subjects design. For the Cambridge and Western propofol anaesthesia datasets, all participants were run in both conditions (awake and anaesthetised) since this was a repeated measures design.

Reporting for specific materials, systems and methods

We require information from authors about some types of materials, experimental systems and methods used in many studies. Here, indicate whether each material, system or method listed is relevant to your study. If you are not sure if a list item applies to your research, read the appropriate section before selecting a response.

Materials & experimental systems

n/a	Involved in the study
☒	☐ Antibodies
☒	☐ Eukaryotic cell lines
☒	☐ Palaeontology and archaeology
☒	☐ Animals and other organisms
☒	☐ Clinical data
☒	☐ Dual use research of concern

Methods

n/a	Involved in the study
☒	☐ ChIP-seq
☒	☐ Flow cytometry
☐	☒ MRI-based neuroimaging

Magnetic resonance imaging

Experimental design

Design type

Resting-state fMRI.

Design specifications

NYU Test-Retest dataset: 3 resting-state scans were acquired. Scans 2 and 3 were conducted in a single scan session, 45 min apart, which took place on average 11 months (range 5–16 months) after scan 1.
HCP Test-Retest dataset: two scanning sessions, separated by 1-11 months.
Cambridge Test-Retest dataset: two scanning sessions, separated by 2–4 weeks.
Cambridge propofol dataset: four scanning sessions: awake, mild sedation (not considered here), moderate anaesthesia, and recovery (not considered here).
Western propofol dataset: four scanning sessions: awake, mild sedation (not considered here), deep anaesthesia, and recovery (not considered here).

Behavioral performance measures

NYU test-retest dataset: no relevant behavioural measures were acquired.
HCP test-retest dataset: behavioural measures unrelated to this study were also collected.
Cambridge Test-Retest dataset: behavioural measures unrelated to this study were also collected.
Cambridge propofol dataset: The level of sedation was assessed verbally immediately before and after each of the scanning runs. Behavioural measures unrelated to the present analysis were also collected.
Western propofol dataset: failure to perform two computerised tasks (a computerised auditory target-detection task and a memory test of verbal recall) was used to evaluate the level of wakefulness in the anaesthesia condition independently of the assessors, who also evaluated participants' level of behavioural responsiveness based on the Ramsay scale.

Acquisition

Imaging type(s)

Functional and anatomical

Field strength

3T for all datasets.

Sequence & imaging parameters

NYU Test-Retest dataset: Each scan was acquired using a 3T Siemens (Allegra) scanner, and consisted of 197 contiguous EPI functional volumes (TR = 2000 ms; TE = 25 ms; flip angle = 90°; 39 axial slices; field of view (FOV) = 192 × 192 mm2; matrix = 64 × 64; acquisition voxel size = 3 × 3 × 3 mm3). For spatial normalization and localization, a high-resolution T1-weighted magnetization prepared gradient echo sequence was also obtained (MPRAGE, TR = 2500 ms; TE = 4.35 ms; TI = 900 ms; flip angle = 8°; 176 slices, FOV = 256 mm).

HCP dataset: for each visit, anatomical (T1-weighted) images were acquired in axial orientation, with FOV = 224 × 224 mm, voxel size 0.7 mm3 (isotropic), TR 2,400ms, TE 2.14ms, flip angle 8°. Functional MRI data (1200 volumes) were acquired with EPI sequence, 2 mm isotropic voxel size, TR 720ms, TE 33.1ms, flip angle 52°, 72 slices.

Cambridge Test-Retest dataset: For each visit, resting-state fMRI was acquired for 5:20 minutes using a Siemens Trio 3T scanner (Erlangen, Germany). Functional imaging data were acquired using an echo-planar imaging (EPI) sequence with parameters TR 2,000 ms, TE 30 ms, Flip Angle 78◦, FOV 192 × 192mm2, in-plane resolution 3.0 × 3.0mm, 32 slices 3.0mm thick with a gap of 0.75mm between slices. A 3D high resolution MPRAGE structural image was also acquired, with the following parameters: TR 2,300 ms, TE 2.98 ms, Flip Angle 9◦, FOV 256 × 256 mm2.

Cambridge propofol dataset: MRI data were acquired on a Siemens Trio 3T scanner (WBIC, Cambridge). For each level of

sedation, 150 rs-fMRI volumes (5 min scanning) were acquired. Each functional BOLD volume consisted of 32 interleaved, descending, oblique axial slices, 3 mm thick with interslice gap of 0.75 mm and in-plane resolution of 3 mm, field of view = 192x192 mm, TR = 2000 ms, acquisition time = 2000 ms, time echo = 30 ms, and flip angle 78. T1-weighted structural images at 1 mm isotropic resolution were also acquired in the sagittal plane, using an MPRAGE sequence with TR = 2250 ms, TI = 900 ms, TE = 2.99 ms and flip angle = 9 degrees, for localization purposes.

Western propofol dataset: MRI scanning was performed using a 3-Tesla Siemens Tim Trio scanner (32-channel coil), and 256 functional volumes (echo-planar images, EPI) were collected from each participant, with the following parameters: slices = 33, with 25% inter-slice gap; resolution = 3mm isotropic; TR = 2000ms; TE = 30ms; flip angle = 75 degrees; matrix size = 64x64. The order of acquisition was interleaved, bottom-up. Anatomical scanning was also performed, acquiring a high-resolution T1- weighted volume (32-channel coil, 1mm isotropic voxel size) with a 3D MPRAGE sequence, using the following parameters: TA = 5min, TE = 4.25ms, 240x256 matrix size, 9 degrees flip angle.

Area of acquisition: Whole brain.

Diffusion MRI: ☐ Used ☒ Not used

Preprocessing

Preprocessing software: Preprocessing of the functional MRI data for both datasets followed the same standard workflow as in our previous studies, and was implemented in the CONN toolbox (http://www.nitrc.org/projects/conn), version 17f [81]. For the HCP dataset, we used the minimally preprocessed data made available by the HCP consortium, with further application of FIX-ICA denoising.

Normalization: Direct normalisation to MNI space (nonlinear) using the segmented grey matter image from each volunteer's high-resolution T1-weighted image, together with an a priori grey matter template.

Normalization template: MNI-152 volumetric template, 2x2x2mm isotropic resolution.

Noise and artifact removal: Denoising followed the anatomical CompCor (aCompCor) method of removing cardiac and motion artifacts, by regressing out of each individual's functional data the first 5 principal components corresponding to white matter signal, and the first 5 components corresponding to cerebrospinal fluid signal, as well as six subject-specific realignment parameters (three translations and three rotations) and their first- order temporal derivatives, and nuisance regressors identified by the software ART 82. The subject-specific denoised BOLD signal time-series were linearly detrended and band-pass filtered between 0.008 and 0.09 Hz to eliminate both low-frequency drift effects and high-frequency noise.
For the HCP data, we used the FIX-ICA denoised data made available by the HCP consortium.
For all datasets, we also compared the effect of global signal regression (GSR) versus no GSR on the resulting network reconstructions.

Volume censoring: For CONN_denoised data, the artifact rejection tool (ART), implemented in the CONN toolbox, was used to identify and regress out outlying volumes, as part of the CompCor denoising procedure described above. The default CONN settings of 5 global signal z-values and 0.9mm were used.

Statistical modeling & inference

Model type and settings: Spearman correlation (two-sided testing) between pipeline properties. t-test comparing PDiv for short-term test-retest versus anaesthesia.

Effect(s) tested: We considered the definition of nodes/brain regions (based on anatomical features, functional homogeneity, or multimodal features); the number of nodes (approximately 100, 200, or 400); four different ways to define network edges (from Pearson correlation or mutual information, each either binary or weighted); and eight different approaches to filter the network's connections (by selecting specific densities, specific thresholds, or using data-driven methods). We also evaluate the effects of a controversial fMRI denoising step, the global signal regression (GSR).

Specify type of analysis: ☐ Whole brain ☐ ROI-based ☒ Both

Anatomical location(s): We considered definition of nodes/brain regions (based on anatomical features, functional homogeneity, or multimodal features, or from group-Independent Components Analysis); we also varied the number of nodes (approximately 100, 200, or 300-400);

Statistic type for inference (See Eklund et al. 2016): We performed network-based analysis (see below).

Correction: We did not perform multiple hypothesis-testing within a dataset, but we repeated our analyses in different datasets to ensure robustness.

Models & analysis

n/a	Involved in the study
☐	☒ Functional and/or effective connectivity
☐	☒ Graph analysis
☒	☐ Multivariate modeling or predictive analysis

Functional and/or effective connectivity	We compared Pearson correlation and mutual ifnormation as methods for quantifying connectivity.
Graph analysis	Our dependent variable was the similarity (portrait divergence) between brain graphs of the same individual across different scanning sessions. We considered both binary and weighted networks, and eight different approaches to filter the network's connections (by selecting specific densities, specific thresholds, or using data-driven methods).
